# Biomimetic polymers and their characterization: toward sustainable materials using synthetic biology

**DOI:** 10.3389/fchem.2026.1846334

**Published:** 2026-07-08

**Authors:** Lumbini P. Ramasinghe, Allan Kenneth Regunton, Michael A. Held, Katherine Leslee Asetre Cimatu

**Affiliations:** 1 Department of Chemistry and Biochemistry, Ohio University, Athens, OH, United States; 2 Nanoscale and Quantum Phenomena Institute, Ohio University, Athens, OH, United States; 3 Molecular and Cellular Biology Program, Ohio University, Athens, OH, United States

**Keywords:** biomimetic polymers, characterization, plant-based materials, sustainable materials, synthetic biology

## Abstract

Biomimetic polymers have emerged as a powerful class of materials that are capable of replicating biological processes, functions, properties, or structures found in natural systems. While extensive reviews are available due to the significant progress in synthetic biomimetic materials, this review focuses exclusively on sustainable, naturally derived biomimetic polymers, as the global focus has shifted towards low-carbon footprint remedies. Biomimicry can only be reliably evaluated using appropriate characterization techniques, and selecting the ideal technique depends on several factors: the specific biomimetic feature of interest, the relevant length scale, the condition of the sample, and whether qualitative or quantitative assessment is required. Hence, we critically survey how complementary chemical, mechanical, structural, and morphological methods are able to span different length scales. We highlight both established and emerging characterization techniques which belong to four broad categories: spectroscopy, microscopy, diffraction/scattering, and thermo-mechanical analysis. Ultimately, this review guides the rational design for next-generation biomimetic materials, bridging the gap between biomimicry, material characterization, and sustainable materials.

## Introduction

**SCHEME 1:**
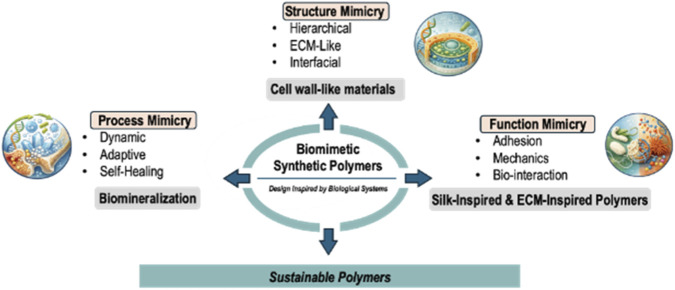
Overview of biomimetic polymer design strategies categorized as process, structural, and functional mimicry, and emphasizes the focus of this review: natural material-derived polymer systems.

Nature has always been an inexhaustible source of inspiration for the design and creation of sophisticated models and architectural motifs (Mundekkad and Mallya, 2025; Zhang et al., 2025). Approximately four billion years of evolution have refined the structures, properties, and processes of living organisms, creating the most efficient structures and systems known. Biomimicry, in its relative infancy since its reintroduction into literature by biologist Beynus (1997), is a field that unravels motifs, patterns, and processes found in living organisms and applies, emulates, and replicates them in the development of new materials, systems, and methods. It is a complex field that is continuously evolving - integrating chemistry, physics, materials science, biology, and engineering at its core. Biomimicry adapts and extracts ideas from nature and encourages the transfer of processes, functions, and structures/forms to develop more sustainable and regenerative materials and systems such as cell wall-like materials, silk-inspired and extracellular matrix (ECM)-inspired polymers, and biomineralization, as a process (Scheme 1).

### Imitation criteria: process, function, and structure

Biomimicry can be implemented by mimicking processes, functions, properties, and forms or structures–individually or in combination. Natural processes can be readily observed and mimicked in living organisms, such as carbon fixation by plants (i.e., photosynthesis) and/or deposition of minerals in crustaceans and mollusks (i.e., biomineralization). By contrast, mimicking functions aim to replicate the operational principles or capabilities of natural systems rather than their external morphology. Restoring, maintaining, and imitating natural functions is the aim of numerous biomimetic studies spanning from materials science to tissue engineering. Lastly, mimicking structures involve translating nature’s designs, assemblies, and hierarchical patterns into engineered materials with enhanced functionality or superior performance. For example, the mosquito proboscis (*Culicidae*) has been an inspiration for the development of jagged and harpoon-shaped needles in the biomedical field to decrease puncture and insertion force of injections with minimal contact to the skin (Aoyagi et al., 2008; Li et al., 2045).

**SCHEME 2:**
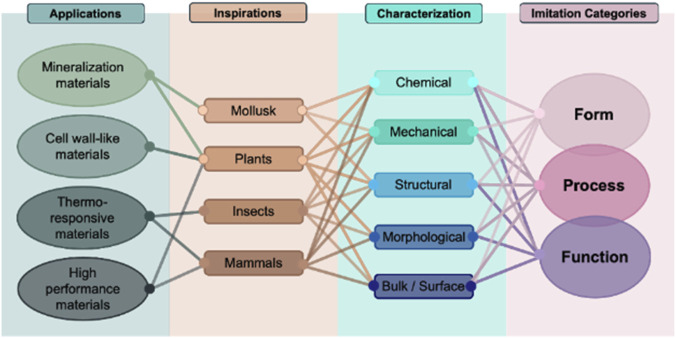
The flow of biomimetic inspiration is influenced by the important aspects: application, biological inspiration, characterization techniques, and imitation criteria.

Scheme 2 provides a visualization of the transfer of properties and/or structures from nature to the materials and processes sought to be mimicked. Application refers to the processes and forms observed in natural systems and living organisms. Imitation criteria can fall into three main categories: processes, functions, and structures/forms. Imitation categories are therefore bridged by biological systems from which inspiration is drawn, highlighting the importance of understanding the underlying biological mechanisms and physico-mechanical mechanisms associated with the applications in mind. This framework establishes a network linking applications, imitation categories, and their corresponding inspirations, with *multimodal characterization techniques* integrated to validate and assess the imitation criteria. As such, this framework emphasizes the mutual reinforcement of the imitation criteria in designing materials, but they can also be viewed independently when a specific design goal is in mind.

Physicochemical and mechanical characterization techniques have proven helpful in guiding the discovery and design of biomimetic polymers. Consequently, strong emphasis has been placed on elucidating the structural details of biological systems, which have been key to the design and generation of true biomimetics. A comprehensive interrogation of natural and biomimetic polymers requires a multimodal analytical approach capable of probing the tripartite aspects of structure, dynamics, and function across multiple length scales. X-ray diffraction (XRD) provides insights into molecular packing and crystallinity at a mesoscale (nm to µm) level, which is crucial for mimicking ordered biopolymer assemblies, such as cellulose microfibrils or collagen fibrils. Solid-state nuclear magnetic resonance (ssNMR) and Fourier transform infrared (FTIR) spectroscopy provide insight into chemical environments, molecular interactions, conformational dynamics, and functional groups at the molecular scale. Thermal analysis techniques provide thermal stability, degradation processes, glass transition, melting behavior, and viscoelastic properties relevant to biological performance in bulk. By contrast, sum frequency generation (SFG) spectroscopy uniquely provides surface-specific molecular organization, which often governs biological recognition at free and buried interfaces. At the nanoscale to microscale, atomic force microscopy (AFM) reveals surface topography, phase separation, and nanomechanics, thus enabling visualization of self-assembly, porosity, and mineralization patterns in biomimetic polymer composites. Collectively, these multimodal techniques at the atomic, meso, and macroscales provide a holistic understanding of biomimicry and offer novel design strategies for sustainable polymers.

### Focus: biomimetic polymers

Biomimetic polymers are a class of synthetic materials designed to replicate the processes, functions, and structures/forms of biological polymers (such as polysaccharides, DNA, and polypeptides) or any other biological systems. Specifically, these polymers are designed at the molecular level to exhibit specific behaviors or properties such as enhanced functionality, adaptability, and performance across a variety of applications. For example, mimicking the hydrophobicity of the lotus leaf (*Nelumbo nucifera*), superhydrophobic electrospun fibrous mats were fabricated, drawing inspiration from micro- and nano-structures found on its surface. The fibers exhibited super hydrophobicity approaching that of the lotus leaf (Lin et al., 2011). Resilin-mimicking materials are another example, which aim to replicate the superior elasticity and high resilience of the insect protein resilin. Synthetic biology has been used to produce recombinant proteins (Balu et al., 2021; Su et al., 2014) or synthetic routes to crosslink resilin with either polyethyleneglycol (PEG) (McGann Cl Fau - Akins et al., 2016) or with (β-[Tris (hydroxymethyl)phosphino]propionic acid (THPP) (Li et al., 2011) to fine-tune the resulting materials with desired properties. In addition to their ability to replicate the complex hierarchical structures and dynamic processes found in natural polymers, biomimetic polymers are attracting attention for their exceptional biocompatibility, biodegradability, and responsiveness to stimuli.

The fabrication of synthetic mimics requires extensive knowledge of the underlying mechanisms and principles that govern biological processes. The challenge lies in translating biological principles into synthetic systems to design, fabricate, and generate advanced and functional materials. In this review, we discuss biomimicry and biomimetic polymers in the context of the three imitation categories (process, function, and property, and structure or form). While extensive reviews discuss synthetic biomimetic polymers, this review focuses exclusively on employing naturally derived/sustainable polymers for biomimetic applications and the potential characterization techniques used to confirm the success of the resulting biomimicry. While extensive literature exists on biomimetic polymers, only a limited number of studies address the characterization techniques used to confirm the success of the resulting biomimicry. Thus, we have chosen to emphasize the crucial role of different characterization techniques in understanding the molecular-level biomimicry of the presented biomimetics. We will describe these as case studies to clearly showcase the importance of these techniques in meeting the specific requirements of a particular application. To highlight each biomimetic, we will discuss in detail the molecular-level features and how they affect macroscopic/bulk properties such as mechanical properties and/or structural forms. We will also highlight plant-based biomimetic polymers, an often-overlooked source of inspiration for the development of biomimetic materials.

## Mimicking a process: biomineralization

Biomineralization is a unique process in which living organisms, such as microbes and plants, can generate and form highly ordered mineral structures via biologically regulated formation of inorganic materials in organic matrices in a precise and controlled manner (Cuéllar-Cruz et al., 2020; Tang and Suzuki, 2023). This natural process enables the formation of hierarchical structures that possess high mechanical strength, biocompatibility, and functionality, as seen in animals (e.g., bones and teeth) and mollusks (e.g., shells and nacre/mother-of-pearl). Biomineralization mimetic polymers regulate the nucleation, growth, and organization of inorganic materials, yielding hierarchically organized organic-inorganic composites with exceptional functional properties, unlocking pathways for innovative biomedical implants/coatings, adaptive structural composites, and environmentally sustainable manufacturing processes. Biomineralization also offers a critical advantage: precise control over nucleation and particle growth at the nanoscale level under the control of various proteins and polysaccharides (Debnath et al., 2024). This allows the process to be tailored at the molecular level, which is not achievable with conventional synthetic methods.

There are two strategies in biomineralization: biologically “controlled” mineralization and biologically “induced” mineralization. Biologically controlled mineralization involves the formation of hierarchically structured, highly ordered, intricate minerals, regulated by interactions between cells and endogenous metals (e.g., cations and anions). Most living organisms harness the synergy between soluble and insoluble components to produce biomolecules with precise, custom-made properties such as organic matrix composition, defined biological functions, crystal growth, etc. On the other hand, induced mineralization is often environmentally mediated mineral precipitation, which occurs indirectly as a result of microbial metabolism and microenvironmental modification. This is observed in microbes (Ronholm et al., 2014) and fungi (Luo et al., 2023) as an indirect result of their metabolic activities that modify the surrounding physicochemical environment, generating local supersaturation conditions. These metabolic changes often change and alter the local environment in which the organisms are found, such as pH, ion concentrations, and redox conditions. This leads to mineral precipitation, which often exhibits significant heterogeneity across various aspects, including variable peripheral morphology, moisture content, crystal structure, trace-element composition, and particle size. Therefore, biologically controlled mineralization is generally considered a direct process, whereas biologically induced mineralization is an indirect outcome of biological activity as a result of interacting with environmental chemistry.

Biomineralization, particularly induced mineralization, can inspire the preparation of novel materials with exceptional functions and structures for biomedical applications, such as bone tissue engineering (Huang D. et al., 2025; Le et al., 2025; Shao et al., 2023; Wang H. et al., 2025) and drug delivery (Tan et al., 2013; Wang et al., 2020). Biomimetic bone regeneration scaffolds would effectively mimic the mineralization process of natural bones. Natural bone constituents can be classified into three categories: organic, inorganic, and water. The organic portion is made from collagen, non-collagenic proteins, and lipids, while the inorganic portion is mainly composed of hydroxyapatite (HAp). HAp, which is a source of calcium phosphate, shows better affinity with bone morphogenetic proteins. A composite that incorporates HAp and a natural polymer can mimic the extracellular matrix of bone, thereby mimicking the bone extracellular matrix. These composites can have the ability to osseointegrate, osseoconduct, and promote new bone tissue formation.

In order to monitor the mimicking of a process, characterization techniques suitable for verifying it are required. In this section, we will discuss various characterization techniques and highlight recent advancements in biomineralization within the context of biomimetic polymers and related composites.

### Scanning electron microscopy (SEM) and energy dispersive X-ray spectroscopy (EDX/EDS)

Scanning electron microscopy (SEM) and energy-dispersive X-ray spectroscopy techniques provide a powerful, complementary toolkit that is useful for evaluating biomineralization in biomimetic scaffolds. SEM is a high-resolution imaging technique that uses a focused electron beam to characterize surface morphology, topography, and microstructural features, while EDX/EDS is an elemental analysis technique to determine the elemental composition and distribution through characteristic X-rays generated by electron-sample interactions. SEM can reveal the surface evolution of calcium-phosphate deposits during nucleation, subsequently forming coalescing leaf-like or cauliflower-like aggregate structures that continue to form apatite layers. SEM images were analyzed to track deposition coverage, particle size, distribution, and layer thickness over the time course of mineralization (Suneetha et al., 2024). Additionally, backscattered-electron imaging of fractured cross sections can show the presence of mineral and organic matrices by contrasting bright mineral regions with darker organic regions, helping determine whether the deposition is superficial or penetrates deeper. Meanwhile, EDX/EDS quantifies the bright mineral domains by mapping Ca and P via Ca/P ratios and detecting other inorganic constituents or contaminants. Hence, the combination of SEM/EDX provides visual, chemical, and semi-quantitative evidence confirming a successful creation of a bone-like material via the biomineralization process (Suneetha et al., 2024; Yadav and Srivastava, 2019). Recent studies use SEM to assess the purity of the inorganic components of the fabricated scaffold composite at a nanoscale level and then monitor the apatite layer formation in order to confirm the quality of the designed material and support its bioactivity (Koshy et al., 2019).

**FIGURE 1:**
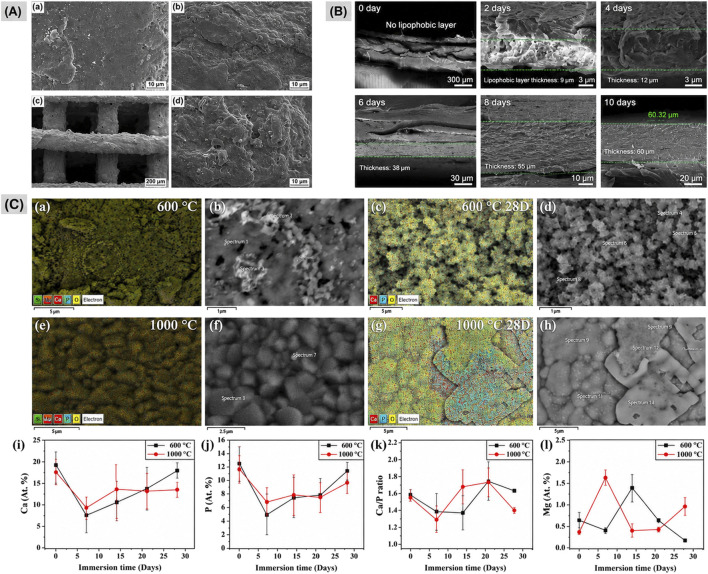
**(A)** SEM images of the scaffolds with the composition 47% HAp/47% PCL/6% gelatin, (a) as fabricated, after immersion in SBF for (b) 14 days, and (c,d) 28 days at two different magnifications (Kadi et al., 2024) **(B)** SEM images showing the time-dependent growth of lipophobic layers at the biomineralized-hydrogel surface (Huang X. et al., 2025) **(C)** SEM images of the morphological changes of natural scaffolds calcined (a,b) at 600 °C, (c,d) at 600 °C in HBSS after 28 days, (e,f) at 1,000 °C, and (g,h) at 1,000 °C in HBSS after 28 days. EDS analysis (i) calcium content, (ii) phosphorus content, (iii) Ca/P ratio, and (iv) magnesium content (Castillo-Paz et al., 2024).

Kadi and co-workers (Kadi et al., 2024) presented a clear progression of biomineralization of 3D-printed HAp/polycaprolactone (PCL)/gelatin scaffolds using HAp nanoparticles synthesized by hydrothermal conversion of *Acropora* coral via SEM imaging. Biomineralization was captured via SEM imaging of scaffolds after immersing in a simulated body fluid (SBF) solution. Figure 1A shows the structure of the scaffold prepared from a 47% HAp/47% PCL/6% gelatin before and after immersion in SBF for 14 and 28 days. Notably, a thick apatite layer was formed after 28 days. As shown in the images (Figure 1A: (c) and (d)), as time progressed, the thick layering of fine particle features became more evident under SBF solution exposure, providing convincing visual evidence of mineral deposition or biomineralization. This suggests that image acquisition of the scaffold surface captured a bone-like mineral coating under matching biomimetic conditions. In addition to SEM results, the authors reported a slight increase in pH in SBF solution, followed by a gradual decrease in pH due to the consumption of OH^−^, and a drop in Ca and P concentrations during apatite formation. This study also used complementary EDX to validate the chemical composition of the synthesized *Acropora* coral-derived HAp nanoparticles and the apatite layer formation. EDX clearly confirms the presence of Ca-P-O through measuring the Ca/P ratio, which was closer to the expected Ca/P ratio of 1.67 value for HAp (Kongsri et al., 2013).

Hung *et al.* (Huang X. et al., 2025) utilized SEM-EDX complementary techniques to convincingly show that alginate hydrogel scaffold formation reproduces key features of natural biomineralized tissues through hierarchical organization, surface-specific mineralization, and bio-interface integration. SEM has provided crucial insights into both surface morphology and biomineralized architecture evolution. The initially smooth surface of the Ca^2+^-crosslinked alginate hydrogel was transformed into densely packed CaCO_3_ nanoparticles upon soaking in a *Bacillus pumilus*-simulated solution. The rice-like morphology of the observed lipophobic layer confirmed the hydrogels’ ability to mimic natural biomineralization behaviors in marine shells and coral exoskeletons (Wei et al., 2015; Zhang et al., 2021). Then, the time-dependent growth of the lipophobic layer upon mineralization was monitored by SEM. As mineralization proceeded for 0, 2, 4, 6, and 8 days, lipophobic layers grew from 0 to 9, 12, 38, and 64 µm thickness, respectively (Figure 1B). The spatial continuity of the mineral layer confirms the strong interfacial bonding between CaCO_3_ nanoparticles and the alginate matrix, consistent with inorganic-organic integrity observed in the bone and dentin biominerals (Lowenstam and Weiner, 1989).

EDX further confirmed the chemical signatures of biomineralization via elemental mapping. The results clearly demonstrate the uniform distribution of Ca throughout the lipophobic layer. The presence of localized Ca at the scaffold surface and its absence in the hydrogel confirmed mineralization at the surface. EDX results supported the authors’ proposed mechanism for the biomineralization process, in which the introduction of free Ca^2+^ in the alginate hydrogel is sourced from the alginate matrix due to a pH increase. This was due to OH^−^ generated by the metabolic activity of *B. pumilus*, which facilitates the formation of a CaCO_3_ layer (Huang X. et al., 2025).

Natural bovine-derived HAp, calcined at 600 °C and 1,000 °C, was employed in scaffold formulation by Castillo-Paz and co-workers to study the effect of calcination temperature on apatite formation. SEM images captured time-dependent apatite formation in Hank’s Balanced Salt Solution (HBSS) after 28 days. The initial smooth surface prior to mineralization was successfully transformed into leaf-like and cauliflower-like apatite crystals upon mineralization. The observed mineral morphology is consistent with the carbonate-substitute apatite in a typical biological system (Figure 1C) (Castillo-Paz et al., 2024).

Similarly, Le *et al.* used SEM-EDX to evaluate biomineralization of chitosan/starch/hydroxyapatite (HAp) composite scaffolds (Le et al., 2025). The SEM images verified the formation of an extensively interconnected, porous network with pore sizes of 150–500 µm. The porous network, necessary for apatite nucleation, facilitates fluid penetration and ion exchange. The progressive formation of spherical apatite clusters under SBF solution confirmed the similarity of the process to biomineralization. Further, EDX confirmed the increase in Ca/P intensities over immersion time, validating the role of HAp nanoparticles, as nucleation sites for apatite precipitation and mineral maturation.

Suneetha and co-workers (Suneetha et al., 2024) designed a modified bacterial cellulose (BC) hydrogel functionalized with phosphate groups. The scaffold is fabricated from a phosphate-crosslinked (bis [2-methacryloyloxy] ethyl phosphate (P)) fibrous BC and polyacrylamide (PAM) hydrogel. Biomineralization was confirmed in SBF, creating a self-assembled bone-like crystalline layer on the fibrous hydrogel matrix after 14 days of immersion. This is quite interesting because, unlike the other biomimetic scaffolds, this innovative composite mineralized in the absence of preloaded HAp. This indicates that phosphate functionalization alone is sufficient to induce nucleation. Similarly, SEM also helped confirm biomineralization of HAp nanoparticles loaded with a gellan gum composite scaffold. EDX further confirmed the secondary mineralization of the scaffold due to the newly formed apatite layer, which contained a similar Ca and P ratio to the HAp (Wang H. et al., 2025).

In all these reported works, SEM and EDX were employed as complementary tools indispensable for verifying the signatures of the biomineralization process at the interfaces. While SEM enables real-time visualization of mineral nucleation, growth, and hierarchical morphological evolution, EDX captures the spatially resolved elemental composition, providing both qualitative and quantitative assessment of mineral identity, stoichiometry, and distribution.

### X-ray diffraction (XRD)

XRD is a robust, non-destructive analytical technique used to investigate crystalline materials. XRD provides comprehensive insights into structure, crystallinity, crystal orientation, phases, and more. Even though XRD is a robust technique, there is a minimum crystal size required for crystals to be considered crystalline; thus, XRD peak widths are inversely proportional to the lateral width of crystallites. The broad crystalline peaks are due to the smaller crystal sizes. Additionally, minor defects can occur in crystalline regions due to the high mobility of surface atoms, lowering the degree of molecular order compared to the bulk of crystallites. To address some of these concerns, several XRD methods are available for calculating the crystallinity index. Depending on the sample type, two X-ray structural characterization techniques are employed: single crystal X-ray diffraction (SCXRD) for well-ordered single crystals, and powder X-ray diffraction (PXRD) using the pair distribution function (PDF) for polycrystalline samples.

*XRD in Biomimetics:* X-ray diffraction techniques, along with complementary methods, have contributed to biomimetic applications. SCXRD was used in structural biology to discover new biomimetic molecules, enabling the identification of atoms in a crystal and their specific locations, along with the electron densities, bond lengths, and angles. For polymeric materials, SCXRD is challenging because they are largely microcrystalline. PXRD provides an alternative technique for assessing structural information of bulk microcrystalline materials. In PXRD, the average diffraction of randomly oriented crystallites yields a plot of the diffracted intensities versus the detector angle, 2-theta (2θ). PXRD can also confirm whether a sample is crystalline or amorphous. In crystalline materials, X-rays are scattered in defined directions, resulting in sharper, well-defined, intense Bragg peaks. By contrast, X-rays are scattered in many directions in amorphous materials because they do not possess periodicity and are ordered primarily at short (two to five Å) and medium-range order (5–20 Å) distances, leading to a broad range of 2θ angles. XRD has been used to determine the size of crystallites using the Debye Scherrer’s equation (Fatimah et al., 2022). Beyond the determination of traditional crystal structure characterization, XRD can be implemented for assessing the degree of biomimicry, as biological tissues and mineralized extracellular matrices possess characteristic mineral phases, crystallinity, crystal size, and structure organization. Hence, XRD-derived information is a critical indicator of successful biomimetic mineral formation in synthetic materials that reproduce the mineral characteristics of their natural counterparts. Natural bone is a hierarchical organic-inorganic composite composed mainly of nanoscale, poorly crystalline, ion-substituted HAp integrated within a collagen-rich extracellular matrix. Hence, XRD-derived information such as phase identity, lattice parameters, crystallinity, crystal size, peak broadening, and preferred orientation can be directly related to the degree of biomimicry.

**FIGURE 2:**
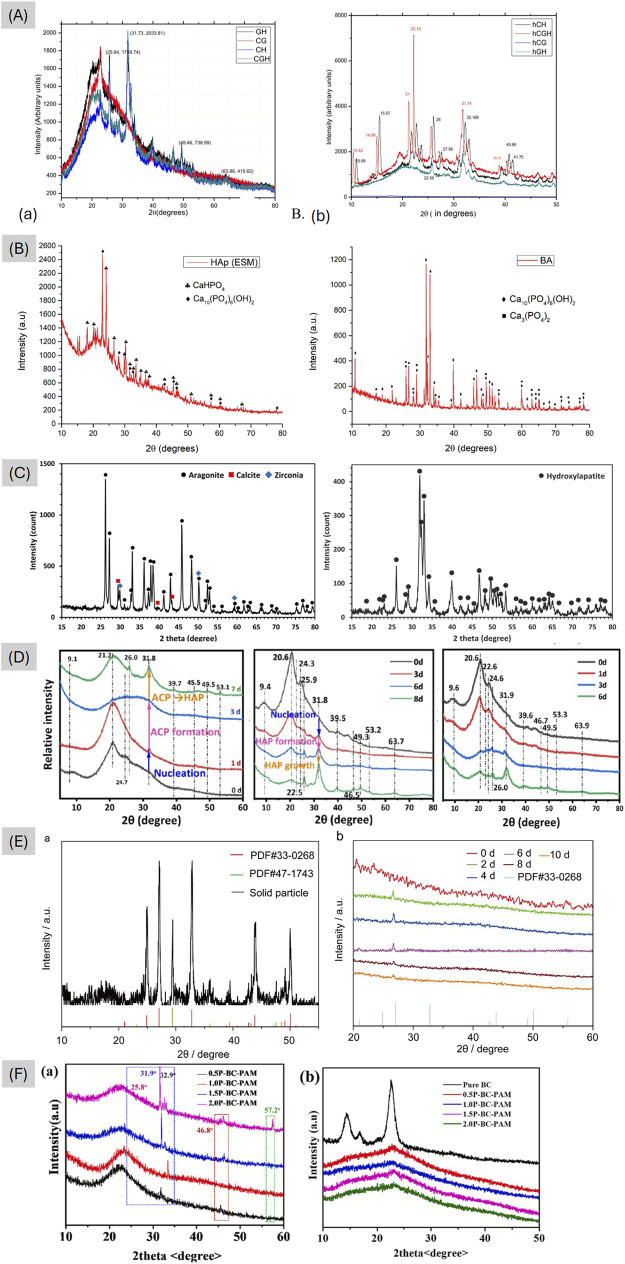
XRD plots of intensity as a function of two theta (2 θ ) **(A)** XRD analysis of composites (a) without osteoblast, and (b) with seeded osteoblast (Yadav and Srivastava, 2019) **(B)** XRD analysis of microwave-synthesized HAp from ESM, and calcined bovine bone ash (Neacsu et al., 2020) **(C)** XRD analysis of ball-milled coral, and HAp synthesized via the hydrothermal method (Kadi et al., 2024) **(D)** XRD analysis of 1-D SNF, 2-D SNF, and 3-D SNF after mineralization at different times (Mei et al., 2025). **(E)** XRD analysis of (a) hydrogels, (b) different ratios of BC-PAM scaffolds before and after mineralization in SBF for 14 days (Suneetha et al., 2024).

*HAp’s Role in Biomineralization:* The crystallinity of HAp plays a critical role in biomineralization by influencing its “bone-like” behavior during biomimetic mineralization. It governs ion release, surface reactivity, defect chemistry, and substitution, all of which can regulate apatite nucleation and growth on the bone scaffold. XRD is commonly employed to evaluate crystallinity, providing insights into composition, lattice parameters, and structural order that are directly related to the materials’ performance in mimicking bone scaffolds. Since the crystallinity of HAp plays an important role in bone scaffolds, PXRD is a robust tool for characterizing HAp structure, crystallinity, and crystallite size. Calcination is a common technique for the extraction of HAp from plant, mammalian, and aquatic/marine sources, as well as from shells and mineral sources (Castillo-Paz et al., 2024; Galeano and García‐Lorenzo, 2014). Phase identity using XRD peaks confirms whether biologically relevant calcium phosphate phases, such as Haps, carbonite apatite, β-tricalcium phosphate, monetite, or whitlockite have formed (Figure 2A). Wang H. et al. (2025) recently fabricated a nanocomposite scaffold loaded with HAp nanoparticles in gellan gum matrix (nHAp-GG) using a freeze-drying technique. They first employed PXRD to confirm the successful synthesis of pure and single-phase HAp nanoparticles within gellan gum-based bone scaffolds and to distinguish this phase from other calcium phosphate compounds, thereby verifying the successful development of bone-like mineral structure. The crystallite size was revealed as 20 nm for HAp by applying the Scherrer equation to the diffraction peak located at 31.8 ° . The scaffold composite was confirmed due to the presence of signature peaks corresponding to the HAp and the gellan gum matrix. The broad peak located at 2θ ≈ 23° was attributed to the amorphous nature of the gellan gum matrix. The presence of HAp diffraction peaks and their similarity to those of native bone apatite provide evidence of successful biomineralization and biomimetic formation. After a week of incubation in SBF, SEM analysis revealed the formation of rod-like apatite layer that covered the scaffold surface, suggesting the occurrence of mineral deposition. However, SEM alone could not determine the crystal structure of the deposited mineral. Since XRD provided critical details by detecting the presence of peaks at 2θ of 25.84, 31.70, 32.90, 34.08, 39.82, 45.40, 46.76, 49.56 ° , assigned to crystal planes denoted by Miller indices (002), (211), (Cannon et al., 2008), (300), (130), (222), (215), and (004) of hexagonal HAp confirmed the successful reproduction of the mineral composition of natural bone tissue mimicking the biomineralization process (Wang H. et al., 2025).

Crystallinity and crystal size are also key indicators of biomimicry. Natural bone apatite is nanoscale and relatively poorly crystalline compared with highly sintered synthetic ceramics. For instance, Galenao *et al.* demonstrated that XRD peak shape is highly sensitive to mineral maturation in bone. This study elucidates the change of pig bone crystallinity upon calcination between 400 °C-1,200 °C (Galeano and García‐Lorenzo, 2014). According to ASTM (American Society for Testing and Materials- 09–0,432, which is equivalent to JCPDS card No. 09–0,432 or ICDD PDF#00–009–0,432), the signature diffraction peaks for HAp correspond to the following diffraction planes of hexagonal HAp: (012), (Ambagaspitiya et al., 2025), (Cannon et al., 2008), (252), (022), (Johnson et al., 1971), (222), and (133) (Neacsu et al., 2020). The results clearly show that the calcination temperature highly affects the properties of the bone samples, producing poor crystalline HAp calcination at 400 °C and 600 °C. The observed color changes were black at 400 °C, olive gray at 450 °C, gray at 500 °C, and grayish white at 550 °C. According to Munsell Color Charts, the grayish white at 550 °C is due to the presence of unburned organic substances. At 600 °C, these organic compounds have disappeared due to a lower content of amorphous phases. At this temperature, diffraction peaks became sharper and narrower due to an increase in crystal size. At 650 °C, all the organic compounds disappeared (consistent with the sample’s white color), and peaks narrowed, resulting in a crystallite size of 28 nm, which then remained stable until 750 °C with no detectable carbonate impurities. With further calcination to 800 °C, intense and sharper peaks were observed, corresponding to an increase in the mineral crystallinity, which is compatible with crystallite growth and elimination of carbonates from the lattice. Above 800 °C, no influence on the crystallinity was noticeable (Galeano and García‐Lorenzo, 2014). Using the FWHM-based Scherrer analysis, the crystallite size ranged from 10 to 17 nm due to the presence of impurities from 400 to 550 °C. At 600and 800 °C, the crystal size increased to 23 and 28 nm, respectively. These results demonstrate how peak broadening and crystallite-size calculations can reveal mineral maturity (Galeano and García‐Lorenzo, 2014).

Other scaffold studies further illustrate how XRD confirms template-directed biomineralization. XRD analysis provides structural evidence for biomineralization in the phosphate-crosslinked bacterial cellulose (BC) hydrogel confirming the formation of HAp upon biomineralization. BC is an excellent scaffold for bone tissue engineering due to its biocompatibility, high surface area, porosity, and mechanical strength. Suneetha et al. used XRD to confirm bone-loke apatite formation after incubation in SBF. Before mineralization, freeze-dried BC scaffolds revealed the presence of characteristic Iα and Iβ phases of cellulose I as evidenced by diffraction peaks at 14.8, 16.6, and 22.9 ° . Upon incubation in SBF, the characteristic peaks corresponding to crystalline HAp were recorded, confirming the formation of bone-like apatite (Figure 2E). The resulting apatite had Ca/P ration 1.75, close to HAp, indicating that phosphate-functionalized cellulose hydrogels can promote mineral deposition resembling bone apatite (Suneetha et al., 2024).

Additional studies further demonstrate how XRD supports the interpretation of biomimetic mineral formation by disguising not only the presence of apatite, but also the mineral pathway and biological relevance of the calcium phosphate phase formed. Neacsu *et al.* developed a biomimetic scaffold using HAp extracted from eggshell membrane (ESM) as bio-template, microwave-synthesized HAp, bovine bone ash, chitosan, and gelatin. ESM was treated in a conventional microwave oven and characterized by PXRD to determine its degree of crystallinity. The PXRD of the HAp-ESM confirmed the presence of not only HAp (Ca_10_(PO_4_)_6_(OH)_2_) but also monetite, aka dicalcium phosphate anhydrous (DCPA, CaHPO_4_). The existence of crystalline peaks corresponding to the diffraction planes (011), (1–11), (210), (−120), and (−123) confirmed the formation of triclinic crystallized monetite. Monetite is a metastable, soluble calcium phosphate phase that can be resorbed *in vivo*, facilitating rapid ion supply and promoting HAp nucleation and growth (Figure 2B). The authors further noted that the diffraction peaks were sharp and intense, indicating well-crystallized calcium phosphate phases, whereas the broad halo at low two theta angles suggested retention of an amorphous organic contribution from the ESM support (Neacsu et al., 2020). The broader importance of phase identification is also supported by the calcium phosphate biomaterials literature. Jeong *et al.* emphasized that calcium phosphates such as HAp, tricalcium phosphate, and whitlockite differ in solubility, stability, ion release, resorption behavior, and biological activity. Therefore, identifying the exact crystalline phase is essential for predicting scaffold bioactivity and bone-regenerative performance. HAp is relatively stable and osteoconductive, whereas tricalcium phosphate (TCP), a calcium salt of phosphoric acid (Ca_3_(PO_4_)_2_), is more soluble and resorbable, and whitlockite is a magnesium-containing calcium phosphate with biological relevance in mineralized tissues (Jeong et al., 2019). Thus, XRD phase analysis directly supports biomimetic assessment because it clarifies whether a scaffold contains stable bone-like apatite, more resorbable precursor phases, or multiphasic calcium phosphate compositions that may better balance osteoconductivity and biodegradation (Figures 2C,D).

Novel 3-D scaffolds mimicking bone structure were fabricated from chitosan/starch loaded with different amounts of HAp nanoparticles (chitosan/starch/HAp) (0.0, 5.0, 7.5, and 10.0% wt./wt. of polymers). A co-precipitation method combined with freeze-drying was used to generate HAp nanoparticles with particle sizes ranging from 60 to 240 nm from chicken bone. The HAp characteristic peaks, along with crystalline chitosan and amorphous starch, were observed in the XRD of the 3-D scaffold. Degradation *in vitro* after soaking the scaffold for 21 days revealed that the peak at 20 ° declined via XRD, which corresponds to the crystalline chitosan in both chitosan/starch/HAp-0 and chitosan/starch/HAp-10 composites. Moreover, upon soaking, the intensity of all HAp peaks in the chitosan/starch/HAp-10 scaffold decreased. The absence of characteristic HAp peaks, including peaks at 2θ = 32.2° and 53.2°, indicates the loss of the initial HAp particles added to the polymer matrix due to scaffold dissolution. These observations emphasize the degradation and mass loss of scaffolds during immersion in the SBF (Le et al., 2025).

All in all, PXRD is a useful, robust, straightforward, and essential technique for characterizing biomineralization in HAp-natural polymer composite scaffolds as discussed above. PXRD simultaneously identifies the formation of bone-like HAp, quantification of phase fractions via Rietveld refinement, which gauge the composition to dissolution and bioactivity, and measurement of crystallinity and crystallite size to gauge the bone-likeness (Scherrer on HAp diffraction planes (002)/(211)). PXRD is also capable of tracking the time-resolved bio mineralization through detecting the transformation of ACP to crystalline HAp, mineral maturation as the HAp peak intensities sharpens and grow.

### Fourier transform infrared spectroscopy (FTIR)

FTIR is an indispensable tool to examine the chemical evolution of biomimetic materials during biomineralization. Mineral deposition upon biomineralization can be validated by identifying the chemical signatures corresponding to phosphate, carbonate, and hydroxyl functional groups which are associated with apatite formation. In bone-mimetic scaffolds, HAp formation during biomineralization is key to the osteoconductivity. Additionally, polymer mineral interactions can also be detected through FTIR during time-dependent mineralization.

**FIGURE 3:**
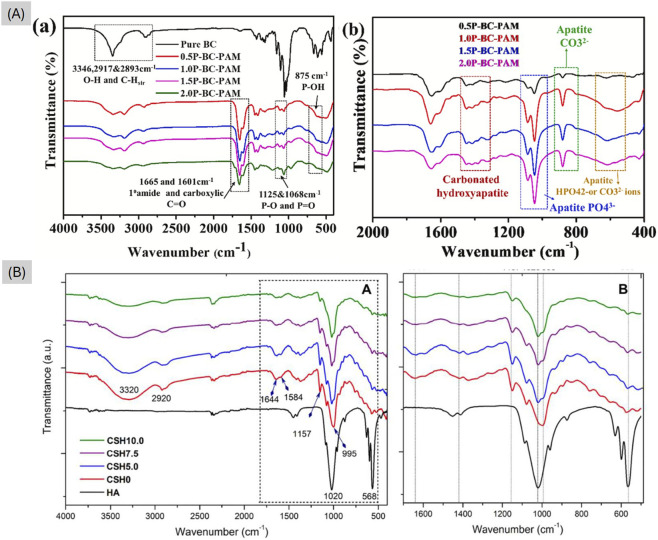
**(A)** FTIR spectra of P-BC-PAM hydrogels (a) before and (b) after 14 days in SBF solution (Suneetha et al., 2024). **(B)** FTIR spectra of (a) four scaffolds containing chitin and chitosan with 0, 5, 7.5, and 10 HAp wt (%) and (b) enlarged spectra from 1700 to 500 cm^-1^ highlighting characteristic peaks of phosphate groups (Le et al., 2025).

*Bovine-derived HAp scaffolds:* Castillo-Paz *et al.* (Castillo-Paz et al., 2024) designed bovine-derived HAp scaffolds, calcined at 600 °C and 1,000 °C to determine the influence of calcination temperature on apatite formation. FTIR spectra revealed the presence of peaks corresponding to the phosphate in HAp at ∼ 1030–1,090 cm^-1^ and doublet at ∼ 565 and 603 cm^-1^ before and after mineralization in Hank’s Balanced Salt Solution (HBSS). Importantly, the scaffold loaded with bovine HAp, calcined at 600 °C indicated the formation of B-type carbonated apatite, which is a mineral phase commonly available in natural bone, due to the presence of carbonate substitution bands at ∼ 872 cm^-1^ and ∼ 1456 cm^-1^. Progressive apatite layer formation was captured through the increasing intensity of phosphate bands, confirms the ion exchange at the scaffold interface leading to strong bioactivity. Unlike in the 600 °C calcination, samples calcined at 1,000 °C show a decreasing OH- band at 3,571 cm^-1^, and almost absent in 28 days spectrum, confirming the dehydroxylation of the scaffold. Similar B-type carbonate apatite formation was observed in hydrogel scaffolds prepared using fibrous BC, acrylamide (AM), and bis [2-methacryloyloxy] ethyl phosphate (BMEP) as a crosslinker through free radical polymerization (P-BC- PAM), designed by Suneetha and co-workers (Figure 3) (Suneetha et al., 2024). Additionally, the authors leveraged the FTIR technique to characterize functional groups in the hydrogel and introduced phosphate crosslinking via the peaks corresponding to P-O stretching at ∼ 1125 and ∼ 1068 cm^-1^, and P-OH functional group at ∼ 875 cm^-1^.

Liu and co-workers (Liu et al., 2025) designed enzymatically mineralized bioactive hydrogel of TEMPO-oxidized (TEMPO- 2,2,6,6-tetramethylpiperidine-1-oxyl) bacterial cellulose (BC) nanofibers and mesoporous silica nanoparticles loaded with angiogenic drug dimethyloxalylglycine into gelatin methacryloyl matrix to mimic the bone extracellular matrix (ECM). The efficient biomineralization was confirmed via the crystalline phosphate (originated from both pre-mineralized nanofibers engineered into the scaffold and from subsequent osteogenic culture conditions) signature peaks presence at ∼560 cm^-1^ and ∼601 cm^-1^ in FTIR spectrum. A notable enhancement of mineralization was observed in the design scaffold due to the peak shifts in carboxylate (∼1,600 cm^-1^) after mineralization, suggesting the chelation of calcium ions by carboxyl groups on oxidized cellulose surfaces.

In order to study the inorganic filler-induced mineralization in biomimetic scaffolds, FTIR was employed by Le and co-workers (Le et al., 2025) for HAp nanoparticle-reinforced chitosan/starch scaffold (Figure 3A). The scaffold composite composition was confirmed by the presence of O-H and N-H stretching peaks at 3,400 cm^-1^ from chitosan, amide I and amide II peaks at 1,650 and 1,550 cm^-1^, respectively, corresponding to polysaccharide backbone, together with phosphate bands at 568 and 1,020 cm^-1^ from HAp (Figure 3B (b)). The increased intensity of the phosphate bands and emergence of the carbonate band confirms the successful nucleation and growth of new apatite crystals in the scaffold after immersion in SBF.

**FIGURE 4:**
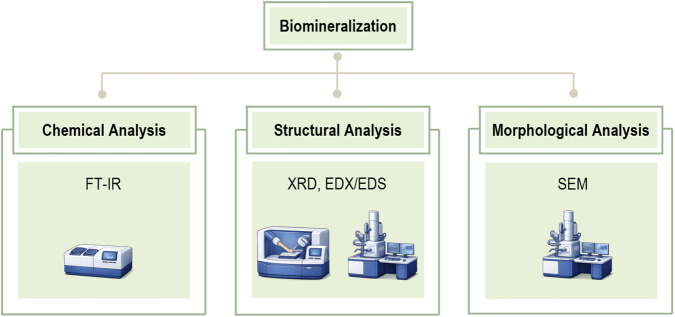
Key characterization techniques to assess biomineralization process in biomimetic composites to detect their chemical, structural, and morphological changes over the course of mineralization. Abbreviations are defined as follows: FTIR (Fourier transform infrared), XRD (X-Ray diffraction), EDX/EDS (energy dispersive X-ray spectroscopy), SEM (scanning electron microscopy).

Thus, FTIR is a critical and main diagnostic tool to monitor molecular evidence of hydroxyapatite for bone-like HAp formation (apatite phase) and the polymer mineral interactions which induce the nucleation on organic polymer matrix during biomineralization in SBF or HBSS environments. Across all the reported studies, FTIR complements SEM/EDX, and XRD (Figure 4) results by providing chemical composition confirmation of apatite biomineralization process in biomimetic bone scaffolds, making it central to evaluating the bioactivity and osteoconductive potential of biomimetic scaffolds.

### Process biomimicry perspectives

The detection of biomineralization in synthetic biomimetic scaffolds requires complementary structural, morphological, and chemical characterization because no single technique can independently confirm the occurrence, composition, crystallinity, and biological relevance of the mineral phases. While SEM provides direct visualization, XRD and FTIR detect chemical identities such as crystalline phases and functional groups of calcium phosphate mineral formation. Reported in several studies, SEM is critical for observing the morphological evidence of biomineralization, including granules, spherulitic particles, rod-like deposits, flower-like crystals, leaf-like structures, or continuous mineral layers on the scaffold surfaces. However, SEM alone cannot determine whether the observed deposits are HAp, amorphous calcium phosphate, salty crystals, or other calcium phosphate deposits. This limitation is critical, specifically incubation on SBF/HBSS immersions. Dried incubated scaffolds can generate artifacts or salt residues that may appear similar to mineral deposits, as SEM requires sample drying and conductive coating, which may lead to shrunken hydrogels, collapsed soft polymer networks, and obscure nanoscale surface features. Moreover, SEM is surface-biased and may not represent mineralization throughout the bulk scaffold. XRD and FTIR provide structural and chemical information in the bulk phase. Since XRD is capable of probing the crystalline phase of deposited mineral is critical in biomineralization. Because the formation of biologically relevant apatite phases with appropriate crystallinity and structural order is key to the characterization of biomimetic mineralization. Despite its importance, XRD carries technical limitations such as insensitivity to thin layer deposition, detecting early stage biomineralization, which can be detected through SEM and FTIR. Moreover, the peak broadening can arise as a result of small crystallite size, lattice strain, defects, etc. Although FTIR addresses some limitations of SEM and XRD, it still has inherent constraints such as interpretive limitations of phosphate bands generated from HAp, amorphous calcium phosphate, monetite, tricalcium phosphate, or other calcium phosphate phases. Hence, FTIR alone is not capable of distinguishing among the mineral polymorphs. Hence, none of these techniques will detect biomineralization on their own; instead, together they allow researchers to determine biomimicry of mineralization.

## Mimicking a function or property: mechanical properties

The mechanical properties of biopolymers dictate how a material responds to forces such as compression, stretching, and resistance to deformation. These properties are key factors used to determine the strength, durability, and flexibility of a material. Biomimetic materials or polymers, inspired by structures and mechanisms found in nature, such as spider silk, have been designed to achieve exceptional mechanical performance. Mimicking the designs and mechanisms by which these structures are made, biomimetic polymers have combined properties that can achieve high strength, high toughness, and adaptive and dynamic functionality that surpasses synthetic counterparts.

The mechanical properties, such as strength, elasticity, resilience, and toughness, determine how a material can effectively withstand external forces without compromising its structural integrity. Strength and elasticity are measured in terms of rates of deformation. On the other hand, the toughness of the material is primarily measured by the Young’s modulus (*E* or *G*). In mathematical terms, it represents the slope of a stress/strain curve (Muiznieks and Keeley, 2013). Biopolymers exhibit elastic moduli that vary significantly, ranging from soft and elastic to stiff and high-strength materials. These mechanical properties are influenced by several factors, including crystallinity, polymer density (molecular weight), chemical structure, copolymerization, and the type and concentration of the filler. Some biopolymers have limited mechanical properties due to weak molecular interactions, which restrict their overall application (Meng et al., 2019). In addition to mechanical properties, studying the thermal behavior of bio-based materials is also a major consideration when designing new materials. The thermal behavior of biopolymers is heavily dependent on the morphology, crosslinking, chemical composition, and the length of the chain. Thus, studies for novel biomimetic biopolymers with outstanding strength has inspired researchers to include thermomechanical properties when investigating biological hierarchies, such as polypeptide sequences found in proteins (silk and elastin), and in the development of high-performance hydrogels that can mimic the extracellular matrix.

**FIGURE 5:**
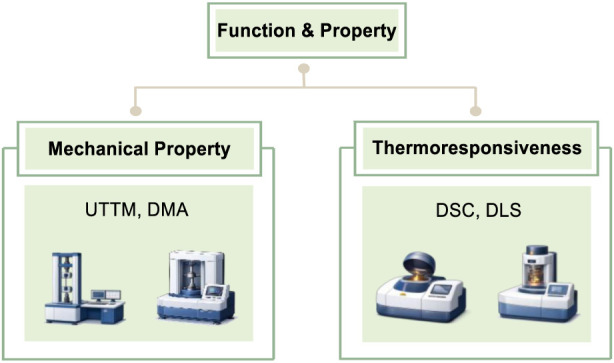
Key characterization techniques to measure mechanical properties and thermoresponsiveness of biomimetic polymers. Abbreviations are defined as follows: UTTM (universal tensile testing machine), DMA (dynamic mechanical analysis), DSC (differential scanning microscopy), DLS (dynamic light scattering).

To verify the mimicked function or property, appropriate characterization techniques to measure these properties are needed. In this section, we review the various characterization techniques used to measure and study mechanical properties of biomimetic polymers and their related composites (Figure 5).

### Silk with tensile strength

Silk is a protein-based material that is gaining broad appeal for its exceptional and highly tunable properties. Spider silk in particular exhibits superior tensile strength with toughness that is on par with synthetic counterparts, such as Nylon and Kevlar (Agnarsson et al., 2010; Graham et al., 2025). Spider silk’s exceptional strength and elasticity are due to its composite structure composed of crystalline β-sheet domains embedded in an amorphous matrix. The crystalline region is composed predominantly of hydrogen-bonded polyalanine (polyA) and polyglycine-alanine (polyGA) block copolymer repeat polypeptides. PolyA motifs form the β-sheet structures, which align to form repetitive backbones that give rise to nanocrystalline β-sheets in the crystalline domain (Trancik et al., 2006; van Beek et al., 2002). These sophisticated molecular alignments are responsible for the mechanical properties of spider silk (Yarger et al., 2018). On the other hand, polyGA repeat polypeptides contain GGX motifs (where X = A, Q, or Y) or GX motifs (where X = Q, A, or R) and are less structured and more amorphous. They assume alpha helical and type II beta turn structures. Compared to polyA, they are less constrained; therefore, they grant spider silk a wider range of extensibility (Yarger et al., 2018). In general, the hard crystalline domain of spider dragline silk gives high strength, while the amorphous region provides toughness and extensibility. Silk from different species has been extensively reviewed by Ramezaniaghdam and colleagues regarding its biomechanical properties, such as stiffness, strength, extensibility, and toughness (Ramezaniaghdam et al., 2022). Here, we discuss characterization techniques used on silk and silk-like materials for biomimicking their functions and properties.

*Universal Tensile Testing Machine (UTTM):* The tensile strength of a biopolymer is known to depend on crystallinity, molecular weight, and the degree of crosslinking. Tensile strength varies from less than 1 MPa to tens of megapascals, depending on the nature of the biopolymer and the processing methods used to develop it. UTTM is used to measure the mechanical input applied to a fiber or material before it fails. It can be used to create stress-strain curves, which provide insights into the mechanical behavior of a material. UTTM analysis coupled with diameter measurements can be used to approximate strength and Young’s modulus, which are critical for understanding how materials can be manipulated to meet specific goals. Here, we discuss how UTTM has been used to characterize the mechanical properties of silk to achieve superior mechanical strength.

In 2010, silk from the orb-weaving spider, *C. darwini* has been identified as the toughest material among silk-producing spiders. These silk fibers were attached to the grips of a tensile tester and pulled until the fibers broke at an extension rate of 10% sec^-1^ with a resolution of 0.1 μm. The force recorded revealed that the silk fibers of *Caerostris darwini* withstood about 1,652 MPa and has a toughness of about 354–520 MJ/m^3^ (Agnarsson et al., 2010).

**FIGURE 6:**
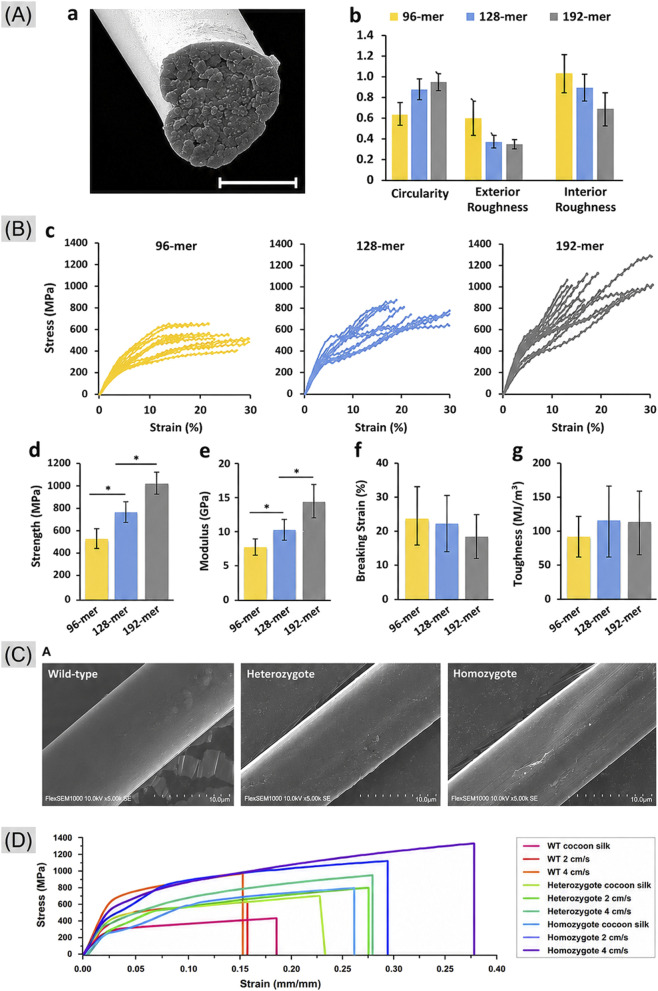
Silk fibers synthesized using a split intein (SI) method **(A,B)** (Bowen et al., 2018) and from transgenic silkworms **(C,D)** (Mi et al., 2023). **(A)** Morphological characteristics of the fibers from the transgenic silkworm: (a) representative SEM micrograph of a 192-mer fiber showing the cross-sectional morphology, (b) circularity, exterior, and interior smoothness of the synthesized 96-mer, 128-mer, and 192-mer fibers **(B)** The compiled stress-strain curves for the synthesized fibers of the 96-mer, 128-mer, and 192-mer fibers shown in (c,d,e,f,g), respectively: (d) ultimate tensile strength, (e) elastic modulus, (f) breaking strain, and (g) toughness. **(C)** SEM micrograph showing the morphologies of wild-type, heterozygous, and homozygous silk cocoons. Stress-strain curves of forced reeled wild-type, heterozygous, and homozygous silks.

In 2018, recombinant spidroins were produced from the iterative construction of a 96-mer repeat from 1 GA-rich repeat motif of *N. clavipes*. Two 96-mer repeats were then combined using split intein (SI)-mediated ligation to obtain a full-length, 193-mer structure (∼556 kDa) (Bowen et al., 2018). The individual fibers spun from the spidroins were characterized using SEM. The obtained morphology and circularity were used to calculate the cross-sectional area (Figure 6A). Axial pull tests were used to measure the tensile strength of the spidroins. Tensile strength was calculated as the maximum load over the cross-sectional area of the initial fiber. Stress-strain curves were generated to obtain the modulus value from the curve slope. Lastly, toughness was calculated as the ratio of the area under the total stress/strain curve and the initial fiber volume. Results showed that the recombinant spindroins matched that of natural silk, in terms of tensile strength (1.03 ± 0.11 GPa), modulus (13.7 ± 3.0 GPa), extensibility (18% ± 6%), and toughness (114 ± 51 MJ/m^3^), making it a true biomimic of naturally occurring silk (Figure 6B) (Bowen et al., 2018).

Silk is not only found in spiders but can also be made by various species of silkworms (*Bombyx* sp.). In 2023, Mi *et al.* (Mi et al., 2023) successfully generated transgenic silkworms that could synthesize whole polyamide spider silk fibers from the fibrin heavy-chain (Fib-H) with a basic structure of Fib-H_12_Fib-L_12_P25_2,_ with fibrin light chain (Fib-L) and a glycoprotein subunit P25_2_). Using CRISPR-*Cas9* gene-editing technology, the Fib-H region was completely replaced with *MiSp*, allowing transgenic silkworms to spin whole spider silks. The silk fibers were subjected to mechanical tests to obtain and calculate the values of stress, strain, Young’s modulus, and toughness. The bionic spider silk exhibited a tensile strength of about 1,299 MPa and toughness of about 319 MJ/m^3^, thus combining properties of ultra-strength and ultra-toughness (Figures 6C,D) comparable to that of a native spider silk from *C. darwini* (Agnarsson et al., 2010).

**FIGURE 7:**
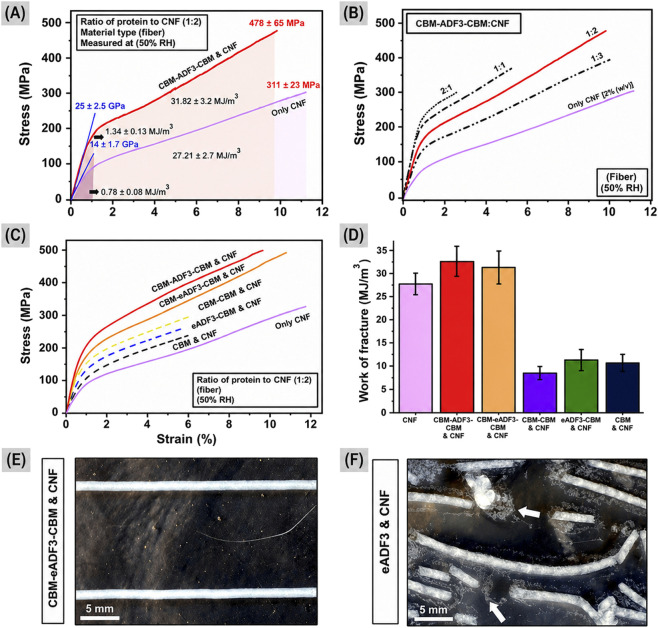
Properties and morphology of the protein-CNF fibers (Mohammadi et al., 2019) **(A)** Stress-strain curves of cellulose-protein fibers of the synthesized triblock spidroins, CBM-eADF3-CBM. **(B)** Stress-strain curve of the fibers spun at different protein:CNF ratios **(C)** Stress-strain curves of the fibers spun with varying compositions of protein and CNF. **(D)** Toughness values calculated from the measured values from **(C) (E)** Extruded fibers (CBM-ADF3-CBM:CNF, 1:2 w:w). Fibers that were collected, which were made with CNF alone or any of the CBM-containing proteins, could be collected in lengths of several meters **(F)** Extruded fibers (ADF3 only, lacking CBM), were fragmented and were not collected as continuous fibers.

Leveraging spider silk’s strength and toughness, composite materials made or derived from silk are another class of high-performance materials that are gaining attention (Chan et al., 2022; Ciulla et al., 2023) In 2019, Mohammadi *et al.* (Mohammadi et al., 2019) combined silk-inspired recombinant proteins to form a tough, fracture energy-dissipating matrix, with cellulose nanofibrils serving as the backbone of the resulting composite. Cellulose nanofibrils (CNFs) were aligned during extrusion to maximize fibril-to-fibril contact, enabling efficient load transfer along the fiber axis. Carbohydrate-binding modules (CBMs) were added to the silk-like proteins (ADF3 and eADF3) to help anchor the CNFs to produce a triblock spidroin protein. The protein-cellulose composites exhibited improved mechanical properties, including toughness (work of fracture), stiffness, strength, and yield point. Increasing the ratios of silk-CBM to CNF from 1:3 (w:w) to 2:1 (w:w) increased the stiffness of the composite from 20 ± 1.3 GPa to 35 ± 6.3 GPa, respectively (Figures 7A–D). In comparison to native spider silk, the fabricated silk composites exhibited superior toughness as measured by tensile strength. Interestingly, as the toughness of the material increased, a decrease in ultimate strain was observed. Higher protein content densified the composite, binding the fibrils more effectively (Figure 7E); however, excessive protein reduced toughness and made the composite brittle (Figure 7F). The study also emphasizes that the content and ratios of the different components can serve as critical parameters for tuning toughness and, by extension, other mechanical properties, to achieve outstanding biomimetic composites.

### Mechanical properties of ECM-like hydrogels

Hydrogels are a class of crosslinked polymers (synthetic or natural) that can retain large amounts of water or fluids in their interstices while maintaining a porous, yet rigid three-dimensional structure, making them suitable mimics of the extracellular matrix (ECM) (Lou and Mooney, 2022). Hydrogels are classified depending on their source, composition, and crosslinking. For example, natural hydrogels are derived from natural polymers, such as polysaccharides and polypeptides (i.e., alginates, collagen, or hyaluronic acid). Synthetic polymers can be prepared through the polymerization of monomers, resulting in polyvinyl alcohol (PVA) (Yu et al., 2025), poly-2-hydroxyethyl methacrylate (PHEMA) (Norioka et al., 2021; Wang Y. et al., 2025), and polyacrylamide (PAAM) (Norioka et al., 2021; Wang Y. et al., 2025), to mention a few.

In terms of composition, hydrogels can be homopolymers, copolymers, semi-interpenetrating (semi-IPN) networks, or interpenetrating (IPN) networks (Dodda et al., 2023). Homopolymer hydrogels consist of only one monomer type, while copolymer hydrogels contain at least two different monomer types. Semi-IPN has polymer networks embedded in a linear polymer chain without the aid of a crosslinking agent. IPN hydrogels are network hydrogels made up of two or more polymer networks that are entangled with each other using a crosslinking agent (Ifergan-Azriel et al., 2025; Muir and Burdick, 2021).

Depending on the degree of crosslinking, hydrogels can be classified as chemically or physically crosslinked hydrogels. Chemical hydrogels are formed through the covalent chemical bonds between individual polymer chains (Milne et al., 2024). Physical hydrogels, on the other hand, rely on noncovalent interactions that hold polymeric chains together, such as hydrogen bonding, hydrophobic interactions, or ionic interactions. Because physical gels exhibit weaker interactions, their mechanical properties are lower than those of chemical hydrogels. Since they have tunable mechanics (in terms of strength and elasticity), hydrogels are being employed for several applications, including, but not limited to, drug delivery (Hashempur et al., 2025), regenerative medicine (Song and Gerecht, 2023), biosensing (Rahman et al., 2025), and even energy storage (Ismail et al., 2023). In addition to their tunable mechanics, numerous efforts have been made to improve hydrogels’ biochemical cues (cell adhesion and spreading) and structural features (such as porosity and alignment). To achieve these, biomacromolecules obtained from natural sources, such as peptides and polysaccharides, are now being modified and incorporated into hydrogel formulations to imitate the complex viscoelastic and dynamic profiles of the ECM found in living tissues. In this section, we will discuss dynamic mechanical analysis (DMA) as a robust method in characterizing the dynamic mechanical environments of gels to mimic natural systems.

#### Dynamic mechanical analysis (DMA)

DMA is a characterization technique that gives insights into the behavior of materials under small changes in temperature or frequency. In DMA measurements, the time- and temperature-dependent deformation or flow characteristics are measured under a given strain. The properties of interest are dynamic modulus and damping coefficients, and key parameters are E′ or G’ (elastic modulus), E” or G” (viscous modulus), and tan (E”/E′ or G”/G′, the damping ability indicating the material’s ability to absorb energy. DMA is used to monitor structural changes and allows us to study the viscoelastic properties of the material being analyzed, including hydrogels.

#### Cellulose-based hydrogels

Among the polysaccharides, cellulose is a promising material that has been utilized in hydrogel preparations. Drawing inspiration from structural orientations in living organisms, hierarchically aligned heterogeneous hydrogels reinforced with the crystalline CNF domains have been made (Xu et al., 2025). The resulting structure is composed of a densified sheath in the outer layer, and an inner aligned porous core. A heterogeneous layer comprising different lengths of fibrillar networks (fibrillar bundles and nanofibrils) is embedded between the inner and the outer layers. The fabricated hydrogel (2 mm) was subjected to tensile tests from which tensile stress and toughness values were calculated. Fracture and fatigue resistance were evaluated using a pure shear test. The generated hydrogel exhibits outstanding mechanical properties, including high toughness (1031 MJ m^-3^), high strength when stretched along the alignment direction (∼55.3 Pa), elongation (3,300%), high stiffness (6.8 MPa), and an excellent fracture energy (552.7 kJ m^-2^) (Xu et al., 2025). In addition to the hydrogel having excellent mechanical properties, the addition of polyvinyl alcohol (PVA) and cellulose nanofibrils (CNF) provided a hierarchically oriented structure mimicking that of the natural muscular tissues (Xu et al., 2025). Moreover, the PVA-CNF gels withstood 10 regeneration cycles, exhibiting outstanding stretchability, getting stronger and tougher with repeated regenerations.

#### Hyaluronic acid hydrogels

Hyaluronic acid is naturally present in the ECM (Leng et al., 2019) and is crucial for structural integrity, hydration, and cellular signaling, including cell adhesion, cell proliferation, and migration. Lou and coworkers (Lou et al., 2018) developed an interpenetrating network (IPN) of hyaluronic acid and collagen hydrogel that is capable of mimicking the viscoelasticity and the fibrillarity of natural ECM which range from 290 Pa (fatty tissues), 668 Pa (connective tissues) (Jamburidze et al., 2017) and as high as 100 kPa for adhesive tissues (Nam and Mooney, 2021). In natural systems, fibrillarity depends on the quantity of collagen present in the cell and the dimensions of the collagen fibrils. These are quantified by measuring the alignment and hierarchical organization of the fibrillar elements using a combination of imaging techniques such as confocal microscopy and atomic force microscopy (AFM) (Doyle et al., 2009; Liu et al., 2024; Sun, 2021).

**FIGURE 8:**
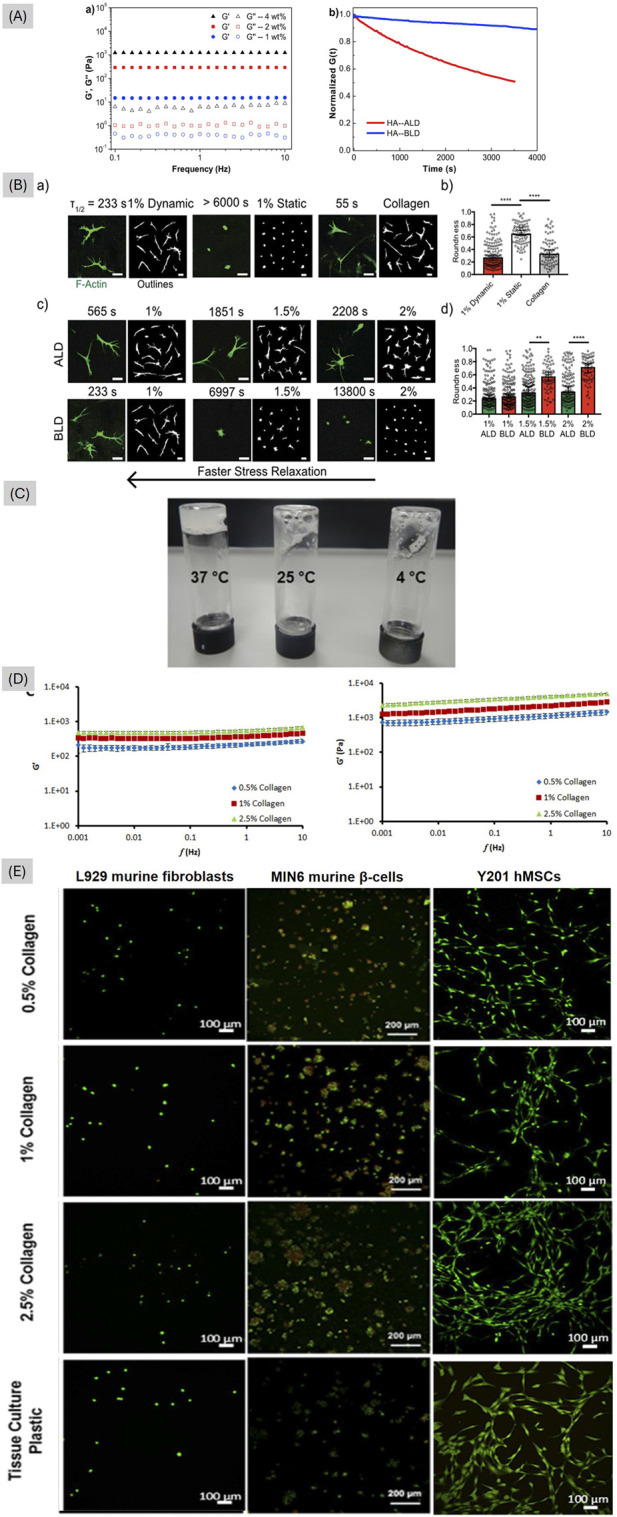
**(A)** Rheological tests of HA SN and HA-collagen IPN hydrogels (Lou et al., 2018). (a) Frequency sweep of HA SN hydrogels at different HA concentrations (HA-ALD), indicating the formation of a stable gel. (B) Hydrazone-structure-dependent stress relaxation of HA SN hydrogels (2% HA) **(B)** The cell morphology grown in the HA-collagen IPNs **(A,B)** and the effect of dynamics crosslinks found in the gel (Lou et al., 2018) **(C)** The fabricated CAF gels at varying temperatures. (D) The graph of elastic modulus (G′) and the loss modulus (G″) as a function of frequency (f) of the CAF hydrogel (0.5%–2.5% w/v) obtained using rheological tests (Montalbano et al., 2018). **(D)** The E’ (G′) as a function of shear strain (γ*) of the hydrogel with varying composition of collagen (0.5%–2.5%) **(E)** The cellular behavior of the different cell lines, L929 murine fibroblast cells, MIN6 β-cell, and Y201 cells, respectively cultured in hydrogels with increasing collagen concentrations (0.5%, 1%, 2.5% w/v).

Rheological measurements, such as frequency sweeps and stress relaxation tests under controlled humidity, were done on the HA-based hydrogel to examine its viscoelasticity and fibrillarity. Results showed that the values of the storage modulus (G′) and loss modulus (G″) were constant at all concentrations, and the ratio of G′ and G″ was >10 for all hydrogel preparations, suggesting the formation of a stable, solid-like network comparable to that of naturally occurring soft tissues (Jamburidze et al., 2017). Additionally, they observed that increasing the concentration of HA to 4% affected the value of the modulus by a hundredfold, emphasizing the role of HA in fine-tuning the mechanical properties of the resulting gel (Figure 8). The hyaluronic acid (HA) hydrogels were modified using aliphatic moieties (HA-ALD) and benzaldehyde (HA-BLD). The aldehyde-modified HA gel relaxed slower compared to the HA-BLD, suggesting a tougher gel (Figure 8A (a and b)). To study the viscoelastic properties of the hydrogel, HA-collagen IPNs were prepared. The collagen concentration was kept at 0.25% (w/v) in all IPN hydrogels with varying HA (1%–2%) to fine-tune the mechanical properties. The IPN hydrogels exhibited a higher modulus (510 ± 58.8 and 733 ± 7 Pa with HA-ALD and HA-BLD, respectively) compared to the prepared collagen - single networks (SNs) hydrogels (collagen-SN), as the controls (57.7 ± 11.5 Pa). This points to a synergistic effect on the role of interactions of HA and collagen in the IPN networks.

To investigate the dynamic covalent cross-linking between the IPN and the HA and/or collagen, in promoting cell spreading and collagen fiber realignment, human mesenchymal stem cells (hMSCs) were used to study the effect of network viscosity on cell morphology (Figure 8). hMSCs encapsulated in 3D HA-collagen IPNs have shown extensive spreading, whereas those encapsulated without the IPN showed round morphologies. Cells that are found in dynamic hydrogels are less rounded compared to those found in static hydrogels or in a pure collagen matrix. The presence of the dynamic environment enhances the cells to interact with each other, thus causing cells to elongate and extend (Figures 8B (a–b)). Comparing the effect of the crosslinking agent on the morphology of the cells, fast-relaxing HA-ALD (HA-aldehyde) and slower-relaxing HA-BLD (HA-benzaldehyde) collagen were analyzed. Increasing the concentration of the ALD crosslinker (1%–2%) leads to slower relaxation of the gel, higher toughness, and more rounded cells. Using the BLD crosslinker, a slower relaxation of the gel makes the cell even more rounded (Figures 8B (c–d)). The generated HA-collagen IPN hydrogel possesses an exceptional viscoelasticity and a fibrillar structure that closely mimics biochemical cues of the ECM such as cell spreading and cell adhesion. The results also suggest that controlling the viscoelastic properties of hydrogels can be a powerful tool for directing cell behavior.

In another study, a three-component hydrogel was fabricated using collagen as an extracellular matrix structural protein, alginate for controlled protein release, and fibrin as a natural regulator of haemostasis and tissue repair (Montalbano et al., 2018). This tricomponent hydrogel (dubbed CAF) was prepared using varying concentrations of collagen (0.5%, 1%, and 2.5% w/v), 5% alginate, and 10% w/v fibrinogen solution. The samples were subjected to rheological characterization. Frequency sweep tests (0.001–10 Hz, strain 0.1%, 37C) were performed. Results showed that all formulations were self-supporting hydrogels (Figure 8C). A 2.5% (w/v) collagen concentration was almost twice as stiff and less swelled compared to the 1% and 0.5% collagen formulations, as expected. Elastic modulus (G′) for all samples remained stable until the strain reached about 1%, with the highest G′ being ∼5 kPa (Figure 8D). The viscous moduli (G″) remained almost constant when the frequency was below 0.1 Hz. Taken together, the CAF hydrogels exhibit viscoelasticity very similar to that of native tissues. Additionally, softer gels (0.5% and 1% w/v) were mimics of softer tissues, such as vascular and dermal-like tissues, providing a homogeneous structure for the cells to perform oxygen and nutrient exchange (Skardal and Atala, 2015).

To assess the behavior of cells, L929 murine fibroblast cells, MIN6 β-cell, and Y201 cells were cultured in CAF hydrogels with increasing collagen concentrations (0.5%, 1%, 2.5% w/v) (Figure 8E). The cells were all viable until the seventh day of culturing. Meanwhile, the 2.5% collagen CAF hydrogel with its stiffer matrix appeared to promote osteogenic activity of Y201 cells, which is more applicable toward structural biomaterial engineering and fabrication.

HA-based and collagen-based hydrogels are promising biomaterials for tissue engineering. However, their weak mechanical properties limit their potential and applications (Brunel et al., 2025). In this regard, new functional groups such as methacrylates and aldehydes are being introduced to form more stable hydrogels, potentially enhancing biomimetic properties and structural properties. Milne *et al.* (Milne et al., 2024) developed a novel dual-modified injectable gel network, made up of aldehyde and methacrylate hyaluronic acid (HA-MA-CHO), that can be used for wound healing and tissue regeneration. The gel network is formed via two separate cross-linking processes. First, a disulfide-containing crosslinker, 3,3-dithiobis (propionic hydrazide) (DTPH) links the aldehyde moieties of hyaluronic acid in a covalent but reversible manner. This leads to the formation of a partially cross-linked hyaluronic acid-aldehyde gel (with G’ ∼500 Pa) that can be injected into tissues. The second step involves a UV-assisted polymerization of the vinyl groups present in the methacrylate groups. Upon irradiation (λ = 365 nm), the soft gel is cured and significantly stiffened, leading to an increase in the strength of the material. The hydrogels were subjected to rheological assessments via time sweep studies at (25 °C). Results showed that before UV irradiation, a single cross-linked network of (HA-MA-CHO) had an approximate G′ of 500 Pa. After subjecting it to UV irradiation, a dual cross-linked network of HA-MA-CHO shown an increase in the storage modulus (G′) to 12.9 and 34.1 kPa for DCN L-HA-MA-CHO and DCN H-HA-MA-CHO, respectively. Thus, a 25-fold–70-fold increase in the G′ compared to the SCN-HA-MA-CHO was observed. This study highlights that modifying the functional groups on the surface of HA, cross-linking, and reaction between HA and other materials can enhance its properties. Moreover, the dual-network hydrogel and its components were found to have a good compatibility with human dermal fibroblasts (NHDFs) until 72 h.

In another study, Li *et al.* (Li et al., 2024) fabricated an ECM-mimetic for bone tissue regeneration. The hydrogel was a composite of interpenetrating polymer networks of gelatin methacryloyl (GelMA) needed for mechanical stability and deoxyribonucleic acid (DNA) required for dynamic capabilities such as cell proliferation and differentiation. The hydrogel composites were also decorated with aptamers to enhance growth factor release and promote the hydrogel as a scaffold for the recruitment of bone marrow mesenchymal stem cells. The composites were analyzed for their mechanical properties. For a hydrogel to be a bone ECM mimic, it must exhibit viscoelasticity and rapid stress relaxation. Based on their results, the composites showed excellent mechanical strength with a G”/G′ ratios ranging from 1% to 33%. In terms of elastic modulus, the GelMA hydrogel (10.94 ± 1.32 kPa) exhibited higher values than the pure DNA-aptamer composite (1.01 ± 0.17 kPa), which was softer and lacked sufficient strength as a scaffold for bone regeneration and repair.

### Elastin-like peptides as thermoresponsive materials

Elastin-like peptides (ELPs) is a broad term used for recombinantly produced proteins inspired by elastin, a naturally occurring structural protein with outstanding elasticity and resilience. Elastin is composed of repetitive motifs of hydrophobic amino acids, VPGX_aa_G, where X_aa_ is any amino acid except proline (Kim and Chaikof, 2010; Urry, 1997). The substitution of the guest amino acid, X_aa_, was shown to have a profound effect on the phase behavior, design, and mechanics of the resulting ELPs, and has been extensively reviewed previously (Chilkoti et al., 2006; López Barreiro et al., 2023). The Pro-Gly moiety increased the propensity of the already disordered peptide to form β-turns, resulting in increased chain entropy and conformational flexibility.

ELPs are amphiphiles, containing a hydrophilic domain and a hydrophobic domain in its core. The hydrophilic domains are rich in lysine residues and are involved in amine-dependent cross-linking to other macromolecules. The hydrophobic domains confer its outstanding elasticity. ELPs are further characterized by their transition temperature (*T*_*t*_), a temperature at which ELPs can undergo reversible phase transitions in aqueous solutions. At a lower temperature than T_*t*_, the protein chain is well solvated (several H-bonding with water) and exists mainly as a random coil. Above the T_*t*_, there is a phase transition that occurs. Water molecules are expelled, and the protein forms internal H-bonding with one another, which ultimately leads to aggregation.

Researchers have manipulated monomeric repeats to generate ELPs with exceptional thermoresponsive properties while maintaining biocompatibility and biodegradability. Several efforts have been made to manipulate the physicochemical and biological properties of ELPs by either changing the composition of X_aa_ or by changing the length of the repeats of the resulting sequence (Rodríguez-Cabello et al., 2018). To be able to monitor the changes in the properties of these ELPs as they are modified, differential scanning calorimetry (DSC) and dynamic light scattering (DLS) techniques are employed. In this section, we will discuss differential scanning calorimetry (DSC) and dynamic light scattering (DLS) in characterizing the changes in these ELPs and how they can mimic natural elastin peptides (Guo et al., 2023).

#### Differential scanning calorimetry (DSC)

DSC is a technique that aids in investigating phase transitions and thermal events by measuring heat flow during heating and cooling events. This analysis reveals the glass transition temperature (T_*g*_), melting points (T_*m*_), and the degree of crystallization of the material. DSC also provides quantitative data on the heat associated with phase transitions. These parameters can provide insights into understanding molecular-level mechanisms underlying hydrophobicity and solvent effects.

DSC has been used to capture energy changes associated with the phase transition of ELPs. Bandiera and coworkers (Bandiera et al., 2023) used DSC to characterize the inverse phase transition properties of two ELPs, a universal ELP (UELP) and a human ELP (HELP). The standard enthalpy (ΔH) of ELPs is associated with the relative hydrophobicity of the polypeptide chains present in the sequence. Thermodynamic analyses performed in Tris buffered saline showed that ΔH for UELP and HELP were 29.0 and 35.0 kJ/mol, respectively, and ΔS for UELP and HELP were 98 and 114 kJ/mol, respectively. Lower ΔH values for UELP indicated it was more hydrophobic and had a higher propensity to adopt beta structures, promoting hydrophobic interactions. DSC was useful in comparing the hydrophobic interaction strengths and the elastin-like phase behavior of the newly synthesized UELP to the native elastin HELP. Moreover, the thermoresponsive properties present in the natural elastin (HELP) were observed also in UELP, though slight differences were observed in the presence of salt and specific designs. In another study, DSC thermal analysis of fiber-forming pentadecapeptides, (VGGLG)_3_, (VGGVG)_3_ and (LGGVG)_3_ revealed that substitution of one amino acid strongly influenced their hydration (Dandurand et al., 2018). The DSC thermogram indicated that a single amino acid substitution led to an increase in the transition temperatures from 55 °C (unordered conformation) to 70 °C, indicating the formation of a more ordered conformation.

#### Dynamic light scattering (DLS) and turbidimetric analysis

ELPs are interesting biomimetic polymers because they undergo phase separation at a lower critical solution temperature (LCST) in aqueous solution. DLS and turbidimetric analyses are utilized to observe this phenomenon. DLS is a technique that is used to measure the hydrodynamic radius and distribution of particles of biopolymers in solutions in the nanometer to micrometer range. It also provides dimensions of the aggregate sizes as a function of temperature. DLS offers real-time monitoring of sudden changes in the morphology or configuration of biopolymers that happen at the phase transition temperature. In tandem with DLS, turbidimetric analysis is also used to observe changes in the morphology or structure of ELPs as a function of temperature. Below the LCST, ELPs are clear and transparent in solution, and as the temperature increases, these recombinant proteins form an inhomogeneous coacervate, –a viscous liquid phase that is immiscible in water, causing the solution to turn from clear to turbid.

The transition temperature of ELPs in pure water is determined by the hydrogen bonding tendency and the number of ELP pentamer repeats (Zhao et al., 2016). As a consequence, the amino acid substitution (X_aa_) significantly influenced the phase transition temperature (Urry, 1997). Incorporation of neutral and hydrophobic amino acids lowered the ELPs’ T_*t*_. On the other hand, the substitution of a charged and hydrophilic amino acids generally increased the T_*t*_. To investigate the effects of repeat length and substitution of X_aa_ to Phe on the self-assembly and thermoresponsive properties of the ELPs, Sumiyoshi *et al.* (Sumiyoshi et al., 2022) synthesized and prepared an elastin-like peptide containing a repeat of (FPGVG)_n= 4 or 5_. Novel truncated ELPs were also generated, containing several repeats of FPGV and/or FPG sequences to study their temperature response and self-assembling properties. The F residue was introduced to maintain the protein’s hydrophobicity, and the PG moiety was conserved for the formation of the β-turn that is implicated in coacervation (i.e., the ability of peptides or polymers to self-assemble under various stimuli, such as temperature). DLS measurements showed that the (FPGVG)_5_ exhibited a 1000-fold increase (3nm – 3 μm) in the diameter above 20 °C. At temperatures higher than T_*t*_, aggregates formed in the solution. The same aggregate phenomenon was observed for (FPGV)_5_ and (FPGV)_4_. Almost all of the ELP analogues prepared exhibited moderate self-assembly ability to produce aggregates above the T_*t,*_ mimicking that of the native elastin (Wang et al., 2019). Interestingly, a shorter peptide repeat (FPGV)_4,_ exhibited reversible temperature-dependent coacervation. In another study, ELPs with X_aa_ = Cys exhibited high apparent viscosity values (Xu et al., 2012). The observed increase in viscosity was attributed to the formation of interfacial cross-linking regions at the sample surface, compared to the bulk of the Cys-ELP solution.

Bandiera and coworkers employed DLS and turbidimetric analysis to characterize UELP and HELP. DLS was performed to measure the changes in the hydrodynamic radius of the biomimetic polymers over a range of temperatures. From the analysis, results showed that below the transition temperature, the volumes of the UELP and HELP particles are small. As the temperature increased and approached the LCST, a sudden increase in the hydrodynamic diameter (D_*h*_) was observed until stabilizing around 300 and 600 nm for UELP and HELP, respectively. Interestingly, both UELP and HELP exhibited a multimodal pattern (starting with small particles to large size particles) over the range of temperatures, as confirmed by the different values of D_*h*_ obtained for both samples. The observed phenomenon is consistent with the observed self-assembly/coacervation of native elastin (Wang et al., 2019).

**FIGURE 9:**
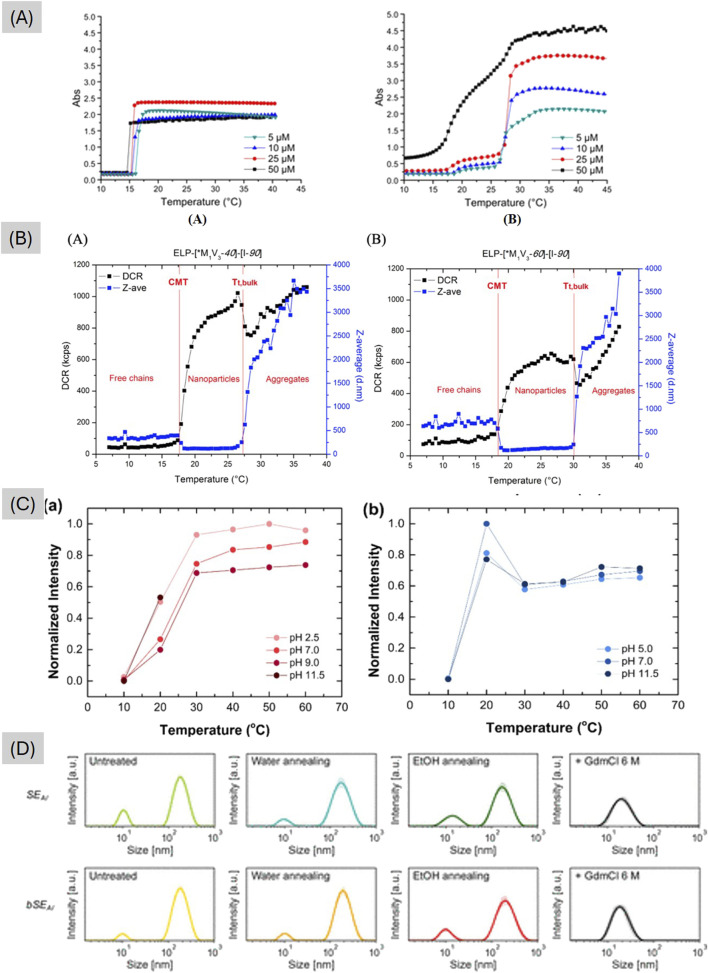
**(A)** Turbidimetric assay of the **(A)** ELP-[M_1_V_3_-40][I-90] and **(B)** ELP-[M*_1_V_3_-40][I-90], the oxidized form **(B)** DLS measurements of the **(A)** ELP-[M_1_V_3_-40][I-90] and **(B)** ELP-[M*_1_V_3_-40][I-90], the oxidized form (89). **(C)** Normalized scattering intensities of the two ELP diblocks, **(A)** K_32_V_64_ and **(B)** E_32_V_64_, under different pH and temperature conditions (10–60 °C) (Choi et al., 2021) **(D)** Size distribution of the different formulations of SELP, SE_AI_ and *b*SE_AI_, measured by DLS under different solvent systems (López Barreiro et al., 2025).

Diblock ELPs are recombinant biopolymers inspired by ELPs that are made up of two distinct elastin-like moieties and joined together in a single polypeptide chain. Each block can have different degrees of hydrophobicity and lengths, which results in a more tunable and precise control over the biopolymer’s properties. Diblock copolymers can form supramolecular assemblies, such as micelles or nanoparticles, with high biological precision and genetic control. Da et al. (2021) refined the design of elastin-like polypeptides to generate them as diblock copolymers. ELP diblocks of the form ELP-[M_1_V_3_-*i*]-[I-*j*] (*i* = *20*, *40*, *60*; *j* = *20*, *90)*, were synthesized. The ELPs have exhibited similar self-assembly characteristics to those of monoblock ELPs. Additionally, modifying the thioether into a sulfoxide (ELP-[M_1_V_3_-*40*]-[I-*90*] → ELP-[*M_1_V_3_-*40*]-[I-*90*]) changes the thermal behavior of the diblocks as was shown in by turbidimetric measurements. The unmodified ELP-diblocks have shown only one sharp transition temperature (17 °C–20 °C), while the sulfoxide modification introduces one additional transition (26–31 °C). Moreover, the thermal behavior of varying lengths of recombinant ELPs, (ELP -[M_1_V_3_-*40*]-[I-*90*] and ELP-[M_1_V_3_-*60*]-[I-*90*]) was evaluated by turbidimetric assay (Figure 9A). DLS measurements revealed that at temperatures below the critical micelle temperature (CMT), scattering intensities are low, suggesting that the ELP diblocks exist as small aggregates (Figure 9B). At temperature between CMT and T_*t,bulk*_, there is a spike in the scattering intensities attributed to nanoparticle formation. Above the T_*t,bulk*_, the recombinant ELP diblocks form aggregates. DLS measurements allow access to different thermal phase transitions of the ELPs with three distinct regimes (unimers, micelles, aggregates). Additionally, the thermal behaviors of the recombinant proteins were analyzed using turbidimetric measurements, and it revealed that the diblock system behaved as an ELP monomer.

To investigate the effect of charged amino acid substitutions on the properties of ELPs, Choi and co-workers (Choi et al., 2021) generated two different diblock copolymers containing glutamic acid and lysine, named [(VPGEG)_32_(VPGVG)_64_] and [(VPGKG)_32_(VPGVG)_64_], respectively. The synthesized ELPs were analyzed using dynamic light scattering (DLS) to gain insights into their self-assembly under various conditions, including temperature, pH, and salt concentrations. DLS measurements of normalized intensities taken from 10 to 50 °C suggested a formation of micellar structures for both K_32_V_64_ and E_32_V_64_ at around 20 °C (Figure 9C). As the temperature was increased to 30 °C, stable monodisperse particles (26–36 nm) were observed under most pH conditions of the analysis. At higher temperatures (under pH 2.5 or 11.5), there are no recorded scattering intensities for both K_32_V_64_ and E_32_V_64_ due to the aggregation of the ELP diblocks in solution. Interestingly, turbidimetric measurements revealed that T_*t*_ shifted to a higher value when the amino acids became cationic or anionic (pH from 2.5 to 11.5). Introducing charged amino acids into the structure of the ELPs makes them more hydrated, more expanded in their form, making them more stretchable, mimicking the elastic behavior of native elastin (Wang et al., 2019). For the thermal behavior of the ELPs, the charged diblock ELPs, there is a shift of the T_*t*_ to a higher value as long as the pH of the solution produces the corresponding ionic forms of the charged moieties. Because of the presence of the charged moieties of these ELP copolymers, they can also form spherical micellar structures that cannot undergo aggregation since there are strong repulsive forces present on the surfaces of these structures.

Recently, ELPs have been conjugated with other biomolecules and biomimetic polymers. This leads to the combination of the biological properties of the biomolecules with the unique and reversible thermoresponsive behaviors of the ELPs. These conjugations result in composites having improved and enhanced properties. ELPs have been conjugated to numerous biopolymers for various applications, including drug release, hydrogel formation, nanoparticle formation, and biosensing. Silk-elastin-like polymers (SELP) are a class of composite materials derived from the combination of silk and elastin. These materials combine the outstanding physical and biological properties of silk and elastin. SELPs can be tuned by the properties of the semi-crystalline silk-like blocks and the stimuli responsiveness and elasticity of the elastin-like blocks. The silk components in SELPs account for the mechanical stability and strength of the composite by forming sheet structures, while the elastin component has a stimulus response when exposed to the specific environment. The balance between the ordered and disordered regions in SELPs was found to determine their self-assembly into hydrogel networks. This is managed by altering the amino acid sequences of the silk and elastin components. Dynamic light scattering was used to measure the hydrodynamic diameter of the different SELPs in solution (0.5 mg/mL) (López Barreiro et al., 2025). DLS measurements obtained a value of 10.9 nm and 11.4 nm for the two different formulations, SE_AI_ and *b*SE_AI_, respectively (Figure 9D). The measured diameters fall within the expected values. However, they note the presence of a more intense peak at higher D_*h*_ values as was previously reported for these types of preparations (Gonzalez-Obeso et al., 2022) in different solvents such as water, ethanol (EtOH), and guanidinium chloride (GdmCl). DLS, alongside other characterization tools, provided an insight into the progression of the self-assembly of SELP into networks. DLS proved useful in determining the state of the polypeptide constructs in the solution, whether they exhibited monodisperse particle sizes or aggregates, before the 3D network formation.

In summary, DSC and DLS techniques both provide complementary information about the performance of a material in terms of its chemical, thermal, and structural behaviors. DSC reveals how a material responds to temperature changes, offering insights into the internal structure and stability of the material. Crucial parameters such as glass transition temperature (T_g_), melting point (T_m_), crystallinity, and thermal stability are given by DSC. DLS, on the other hand, gives us insights into the particle size and distribution of the material. It also offers insights into the stability of the material over time, most especially the uniformity of the particle sizes. Utilized together, they are powerful techniques in interrogating the structural features at the nanoscale level and connecting them to the thermal behavior at the macroscopic level.

### Functional biomimicry perspectives

The reported studies here were successful in replicating the mechanical and dynamic properties of natural biomaterials. However, direct comparisons remain difficult due to the differences in testing conditions and characterizations used in these studies. Variations in parameters such as humidity, strain rate, sample preparation, fiber morphologies directly influence the measured strength, extensibility, and toughness of the generated materials highlighting the need for standard and uniform protocols for testing. Current characterization schemes often rely on single techniques, which may not fully capture viscoelasticity and fatigue resistance. Additionally, measurements are frequently performed under controlled environments that do not fully recapitulate the complex biochemical and mechanical conditions encountered *in vivo* or under physiological conditions. Mechanistically, the exceptional properties of biopolymers arise from the unique and hierarchical organizations among the different domains present, allowing adaptability and mechanical resilience. Finally, advances in synthetic biology have accelerated the development and generation of biomimetic materials using the design of recombinant polymers (such as silk) with tailored functionalities. Future research directions should integrate emerging synthetic biology in tandem with multiscale characterization in establishing a more comprehensive structure–property relationship of the biopolymers to develop adaptive and intelligent materials, and not merely replicating those found in natural systems, for a variety of applications.

## Mimicking a structure: plant cell wall

Mimicking a structure, rather than simply reproducing a chemical composition, is an arduous but powerful emerging strategy in the fields of synthetic biopolymers. The plant cell wall is an extraordinarily optimized hierarchical architecture that has been endowed by nature. Plant cell wall structures have garnered attention for their multi-scale structural organization, mechanical anisotropy, control of interfacial polarity, and supramolecular assembly. Even though there are structure-inspired material designs that span biomineralized tissues, ECM environments, and protein assemblies (as discussed above), there is limited progress on sustainable polymer systems that successfully mimic their natural counterpart architectures beyond plant cell wall composites. In this portion of the review, we focus on plant cell walls and plant cell wall mimetic composites. We highlight previous attempts at structural mimetic assemblies and, most importantly, emerging characterization techniques that study nanoscale ordering, chemical functionality, molecular alignment, and dynamic interfacial interactions, which are important for the design and generation of novel, low-carbon footprint, biomimetic materials.

**FIGURE 10:**
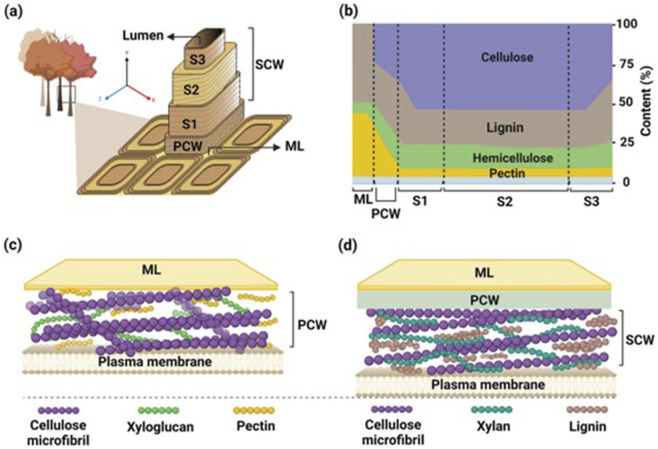
Plant cell wall architecture and composition. **(a)** A cut-out schematic of a wood cell wall shows its layered structure, featuring a network-like arrangement in the PCW and aligned fibrils in the SCW (S1, S2, S3- sublayers of SCW). **(b)** The composition of wood cell walls in each layer after lignification. The light blue region shows the content of other compounds in the layers. **(c)** PCW schematic based on a molecular model, depicting the cellulose-hemicellulose composite embedded in a matrix of pectin and structural proteins (not shown). **(d)** A schematic of the SCW (deposited interior to the PCW), which contains significant lignin deposits surrounding the cellulose network (Koshani et al., 2025).

**FIGURE 11:**
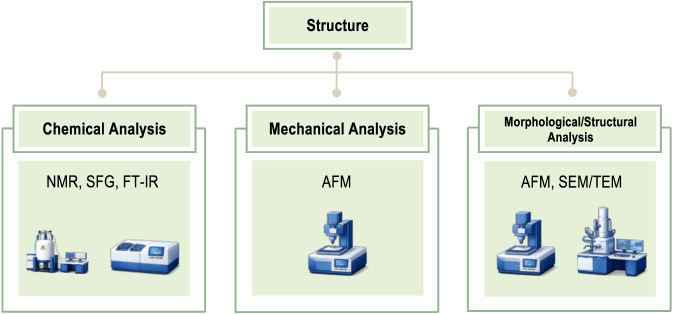
Key characterization techniques to assess plant CW structures to detect their chemical, mechanical, and morphological analysis. Abbreviations are defined as follows: nuclear magnetic resonance (NMR), sum frequency generation (SFG), Fourier transform infrared (FTIR), atomic force microscopy (AFM), scanning electron microscopy (SEM), transmission electron microscopy (TEM).

Plant cell walls are complex composite materials consisting of carbohydrate polymers (including cellulose, hemicellulose, pectins), structural glycoprotein polymers, and aromatic polymers (i.e., lignin) (Figure 10). The composition of these polymers varies markedly across different plant cell types and organs, reflecting their synergistic, functional roles in plants. For example, primary cell walls (PCWs) are thinner, more flexible walls synthesized during cell growth and expansion. PCWs are composed mainly of paracrystalline cellulose microfibrils that are coated with various hemicellulose polymers and are embedded in a gel-like glycoprotein and pectin matrix. Matrix components help facilitate wall elongation, porosity, and ion exchange (Cybulska et al., 2010; Kozioł et al., 2015). Cell-cell adhesion is mediated by the middle lamella (ML), which consists mainly of hemicellulose and pectin polymers, but also contains a significant amount of aromatic lignin polymer. Secondary cell walls (SCWs) are wall thickenings deposited after cell expansion, and are comprised mainly of crystalline cellulose polymers that form tightly packed, highly oriented microfibrils to confer tensile strength. Hemicellulose and pectins are reduced in SCWs, and are instead replaced by lignin polymers, which impregnate the cellulose network to provide rigidity, hydrophobicity, and decay resistance (Cybulska et al., 2010; Dupree et al., 2015). These structures have been characterized to reveal their chemical, structural, and mechanical characteristics at the micro and nano levels as summarized in Figure 11 (Koshani et al., 2025). In this section, we will review various biophysical characterization techniques used for plant cell walls spanning the micro to nanoscales.

### Nuclear magnetic resonance (NMR)

Nuclear magnetic resonance (NMR) is emerging as a powerful tool for detecting the molecular structures and dynamics of natural cell walls, and cell wall mimetic composites. Solid-state NMR (ssNMR) is superior to solution-state NMR or chromatographic analysis owing to its ability to probe intact, unprocessed CWs, preserving their native architecture, whereas other techniques require solubilization or chemical extractions. This serves as a powerful tool that integrates three approaches, namely, cross-polarization (CP), magic angle spinning (MAS), and high-power coupling (Dupree et al., 2015; Foston, 2014; Kang et al., 2019; Koshani et al., 2025). The contribution of each technique can be broken down as follows. CP can overcome the weak signal of ^13^C by transferring magnetization from protons to carbon, thereby increasing the ^13^C sensitivity and reducing dipolar interactions. MAS allows the sample to spin at an alleged magic angle to understand the effect of the anisotropy of the solid sample. There are several NMR techniques for analyzing cellulose crystallinity, including C4 peak separation, subtraction of standard amorphous cellulose/amorphous subtraction, and average lateral microfibril size (L) or crystallite size methods. ^13^C CP/MAS NMR spectrum of natural cellulose shows broad resonances that spread over chemical shift ranges of 101–108, 80–92.5, and 57–67 ppm assigned to the C1, C4, and C6 carbons of cellulose, respectively.

There are pioneering investigations employing the 1-D, 2-D, and 3-D ssNMR for qualitative and quantitative analysis of how cell wall constituents of cellulose, hemicellulose, pectin, and lignin are spatially organized and their interactions in intact PCW and SCW. The key studies worked on native plant cell walls of *Arabidopsis thaliana, rice, maize, switchgrass, and Arabidopsis plant etc.*, have shown that usability of ssNMR to reveal the cellulose-hemicellulose, lignin-polysaccharide, water-polysaccharide interactions that underpin cell wall architecture and mechanisms (Dupree et al., 2015; Hill et al., 2009; Kang et al., 2019). These studies established the importance of performing ssNMR to investigate the cell wall architecture, interaction motifs, and dynamic hydration mobility, thereby obtaining a comprehensive understanding of native plant cell walls and enabling the design of plant cell wall mimetic polymers.

**FIGURE 12:**
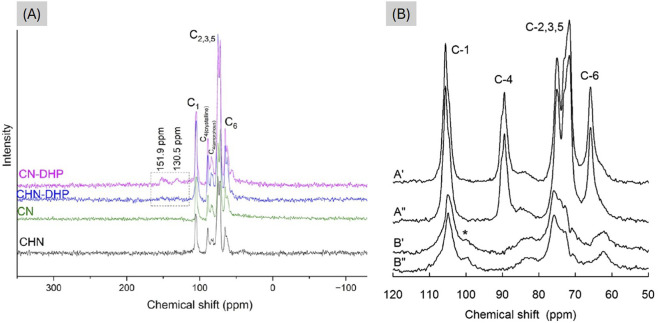
**(A)** ^13^C CP/MAS ss NMR spectra of dried cellulose−hemicellulose nanofibrils (CHN), cellulose nanofibrils (CN), cellulose−hemicellulose nanofibrils with lignin (CHN-DHP), and cellulose nanofibrils with lignin (CN-DHP) (Snyder et al., 2025), and **(B)** Proton spin relaxation editing experiments subspectra separated from the normal CP/MAS spectrum of the composite of bacterial cellulose and tobacco ^13^C- XG by exploiting differences in: A′, B′ -proton rotating-frame spin relaxation; A″, B″-proton spin-spin relaxation. Carbon numbers refer to glucosyl residues of cellulose. A star indicates a signal assigned to C-1 of xylose residues in Xyloglucan (Bootten et al., 2009).

Snyder *et al.* (Snyder et al., 2025) developed a synthetic plant SCW from cellulose-hemicellulose nanofibrils alongside *in situ* lignin polymerization. They employed ^13^C CP/MAS NMR to play a dual role: one is to confirm the preservation of cellulose crystallinity during enzymatic hemicellulose removal, and the other is to probe the successful incorporation of lignin into the polysaccharide scaffold. ssNMR analysis reveals the nearly identical cellulose crystallinity for both cellulose and cellulose-hemicellulose substrates, confirming that the observed differences in lignin deposition are consequent of the presence or absence of hemicellulose, not because of cellulose structural alteration. The characteristics of lignin spectral features were detected, revealing that lignin was successfully incorporated into the insoluble fibrillar network (Figure 12A). This is a key feature in biomimetic lignocellulosic systems, which is unable to be detected through solution-state NMR because immobilized lignin fractions are unable to extract into the solvent.

A pioneering study led by Bootten and co-workers (Bootten et al., 2009) synthesized a model composite to mimic PCW using cellulose from the bacterium *Gluconacetobacter xylinus* and ^13^C-labeled xyloglucans from the walls of a tobacco plant. They implement ssNMR with cross-polarization (CP) and magic-angle spinning (MAS) to investigate possible molecular interactions between xyloglucans and cellulose in plant cell walls. Xyloglucan signals were found in less rigid domains, and no signal was detected from the cellulose crystallites. The chemical shift observed for the Xyloglucan C1 resonance from 103 ppm in solution to 104.8 ppm in the solid state, alongside C4 displacements to 81–84 ppm reflects the transition of Xyloglucan from mobile coil to semirigid conformation (Figure 12B). This is a pioneering study that establishes a precise architecture for synthetic plant cell wall mimics to reproduce. A composite structure in which cellulose crystallites were embedded in a matrix of Xyloglucan with a semirigid (straightened backbone) conformation, that is, a matrix that is partly ordered rather than amorphous.

### Sum frequency generation (SFG) vibrational spectroscopy/microscopy

SFG vibrational spectroscopy offers a unique and powerful approach for studying plant cell walls, as it can probe the non-centrosymmetric crystalline domains without masking contributions from the amorphous polysaccharides in the cell wall composite. Unlike other vibrational spectroscopies, SFG has chemical selectivity and provides conformational and structural information on crystalline cellulose microfibrils, enabling direct visualization of cellulose microfibrils (CMF) assembly and organization in intact plant cell walls (Barnette et al., 2011; Lee et al., 2014; Makarem et al., 2019; Park et al., 2013). Several studies focus on CMF structure and orientation in native plant cell walls. Hence, SFG can serve as a benchmarking validation tool for polymer-based plant cell wall mimetic structures, which aim to reproduce PCW and SCW structures. In this section, we will introduce the use of SFG for probing the structural conformation of plant cell walls and discuss its applications. The same approach can be applied to validate controlled stereoregularity and to determine the SCW analogs using signals from the non-centrosymmetric crystalline domains of biomimetic polymers.

**FIGURE 13:**
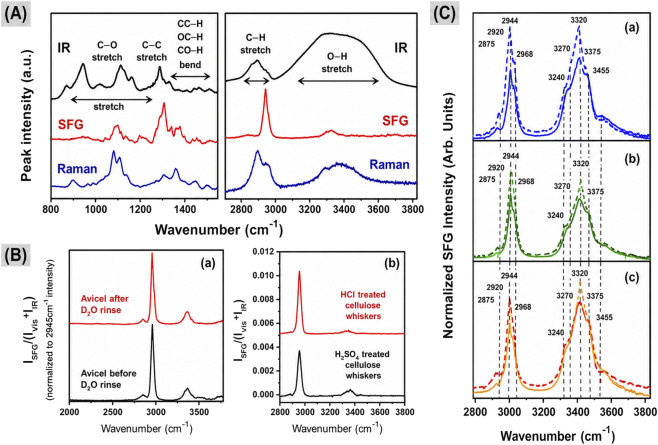
SFG characterization of plant cell walls **(A)** Comparison of IR, SFG, and Raman spectra of microcrystalline cellulose taken in ambient air (Barnette et al., 2011) **(B)** SFG spectra of cellulose (a) before and after D_2_O exchange, and (b) H_2_SO_4_ and HCl-treated cellulose whiskers (Barnette et al., 2011) **(C)** SFG intensity for (a) the native cellulose, (b) xyloglucan-cellulose, and (c) glucomannan-cellulose composites and pretreated counterparts (dashed lines) (Shah et al., 2018).

Barnette *et al.* (Barnette et al., 2011) laid the conceptual basis for extracting crystalline cellulose information from heavy interference and spectral overlap caused by amorphous signals using SFG. Since SFG generates signals from the non-centrosymmetric vibrational modes in crystalline cellulose, all the other amorphous constituents, including hemicellulose and lignin, are unable to generate SFG signals. The SFG, IR, and Raman spectra of crystalline cellulose model samples were first compared. As shown in Figure 13A, SFG spectra contained sharp peaks at 2,850, 2,945, and 3,325 cm^-1^ corresponding to CH_2_ symmetric, CH_2_ asymmetric, and O-H stretching vibrations, respectively, while IR and Raman spectra exhibited broad, congested CH/OH signals. The authors used woody biomass samples such as oak, birch, and pine for comparative SFG, IR, and Raman analysis. The SFG spectrum is identical to that of pure crystalline cellulose in Figure 13A. They then extended their work to observe the modification of surface OH groups via H/D exchange to identify exchangeable hydroxyl groups and crystalline cellulose from disordered polysaccharides and water, as well as H_2_SO_4_, and HCl-treated cellulose whiskers for to selectively disrupt crystalline cellulose and observe signal disappearance accordingly. When amorphous cellulose is selectively disrupted, the resultant SFG signal is attenuated or diminished. These chemical perturbations demonstrate that the SFG signal depends on the crystalline order rather than the mere presence of cellulose microfibrils. As shown in Figure 13B, SFG spectra are identical before and after D_2_O exchange and acid hydrolysis, revealing that the SFG signal originates from the bulk crystalline ordering inside the cellulose microfibrils because only highly ordered, non-centrosymmetric crystalline cellulose result active SFG O-H vibrational signals (Barnette et al., 2011).

SFG has emerged as a unique spectroscopic tool for studying *in situ* cellulose organization. SFG selectively reports non-centrosymmetric crystalline cellulose, suppressing the signal from the amorphous matrix, while IR, Raman, and NMR techniques are obstructed by amorphous matrix interference, and XRD loses resolution at the nanoscale packing regime. SFG can track polar cancellation, I α /I β polymorph distribution, lateral aggregation, and matrix-induced packing modulation at the nanoscale, revealing structural hierarchies that were previously inaccessible inside the intact plant cell walls (Lee et al., 2014; Park et al., 2013; Park et al., 2014). Park et al. (2013) monitored the mesoscale ordering of cellulose in intact plant cell walls. They confirmed that SFG is sensitive to microfibril alignment and packing of cellulose within the cell wall. SFG peak located at 2,944 cm^-1^ corresponds to the crystalline cellulose in *Arabidopsis thaliana*, while changes in the 3,320/2,944 cm^-1^ intensity ratio suggest subtle changes in cellulose ordering as tissues mature. The peak at 3,320 cm^-1^, which is attributed to the O-H signal, is highly sensitive to coherent hydrogen-bonded crystalline structure, while 2,944 cm^-1^ corresponds to C-H vibrations primarily reflects the presence of cellulose. Hence, this ratio allows to selective measure the meso-scale cellulose organization.

The pioneering works paved the early investigations of employing SFG to detect the orientational order, crystallinity, mesoscale assembly of cellulose microfibrils for native plant cell walls. These studies established essential structural reference points for cellulose organization in cell walls by defining spectral fingerprints and orientational order for crystalline cellulose across different plant tissues and bacterial cellulose films (Lee et al., 2014; Lee et al., 2023).

Shah et al. (2018) demonstrate a cell wall mimetic model of hemicellulose−cellulose composites that mimic plant cell wall polymer interactions. The model was prepared by synthesizing deuterated bacterial cellulose in the presence of glucomannan or xyloglucan. They employed SFG with complementary X-ray and neutron scattering data to study how different hemicelluloses influence the structural organization and stability of crystalline cellulose in the plant cell wall. SFG probes crystalline cellulose within the composite and changes in SFG signal intensity and spectral features, whether cellulose ordering is preserved, reorganized or disrupted with a change in hemicellulose composition. The peaks at 3,240 and 3,270 cm^−1^ are characteristic of the presence of cellulose Iα and Iβ, respectively (Figure 13C). By deconvolution of these two peaks, the relative amount of cellulose Iα and Iβ allomorphs in cellulose and composites was estimated. Upon dilute acid pretreatment Iα/Iβ ratio remains the same in hemicellulose−cellulose composites. This reveals that interactions between the xyloglucan and glucan chain can alter the allomorph of the crystallite in the microfibrils. Moreover, since glucomannan remains the same, it reflects that glucomannan does not directly interact with the glucan chain during cellulose microfibril formation.

The pioneering studies on native plant cell walls clearly indicate that SFG spectroscopy and microscopy can be employed as versatile tools that bridge the fields of plant biology and materials science. These techniques can be further extended in the next decade toward plant cell wall structural mimicry for synthetic, biomimetic polymer designs.

### Atomic force microscopy (AFM)

AFM is a widely employed technique for nanoscale visualization of plant cell walls and wall-like biomimetic composites, owing to its cost-effectiveness and minimal sample preparation as compared to other microscopic techniques, such as SEM and TEM. The key requirements to mimic a plant cell wall are that it reproduces (i) the compositional hierarchy (cellulose scaffold + hemicellulose tethering + pectin matrix), and (ii) structural hierarchy possess through specific interactions between compositional components.

**FIGURE 14:**
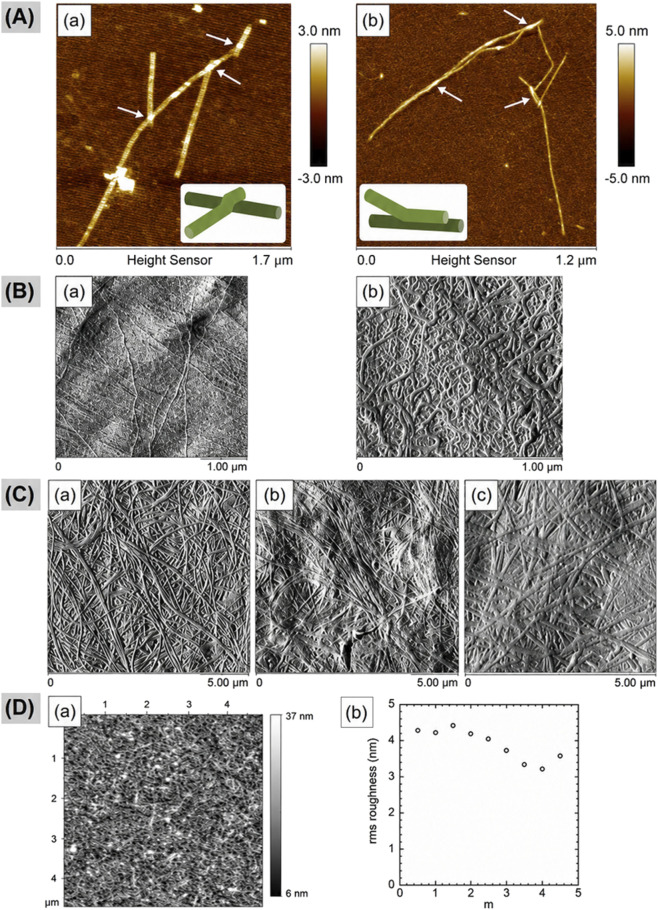
**(A)** Xyloglucan chain assemblies. (a) end to end connection of a molecule to another. (b) surface adherence assembly (Kozioł et al., 2015), and **(B)** AFM topographies (a) reconstituted apple cell wall material prepared by means of the buffer-phenol method (CWMR), **(B)** fixed apple cell wall (FAT) (Cybulska et al., 2010) **(C)** AFM topographies of **(A)** BC, **(B)** BC embedded in pectin, and (c) BC with pectin and xyloglucan created by adding the apple pectin (Cybulska et al., 2010). **(D)** (a) AFM topography, and (b) rms roughness of cellulose nanocrystals and xyloglucan assembly (Jean et al., 2009).

A detailed assessment of surface morphology, molecular conformation, and assembly behavior of plant cell wall polysaccharides such as cellulose and hemicellulose has been collected using AFM. A key hemicellulose component, which is responsible for crosslinking of CMFs in the PCW, is xyloglucan (XyG). Koziol and co-workers (Kozioł et al., 2015) employed AFM to evaluate the nano-structure and self-assembly of XyG extracted from tamarind seed (*Tamarindus indica L.*) on a mica surface. Topographic imaging in ambient butanol revealed rod-like morphology of individual XyG chains with a mean molecular height and length of 2.3 ± 0.5 nm and 640 ± 360 nm, respectively. Further, XyG molecules exhibit a twofold helical structure with an approximate periodicity of 115.8 ± 29.2 nm. The helical undulations and the bending regions were confirmed by the height profile along the molecular skeleton. Cross-like, parallel-like, and rope-like self-assembly of XyG were also observed as shown in Figure 14A. These assemblies show XyG’s ability to self-organize into ordered supramolecule architectures. These arrangements confirm the mimicry of interactions between XyG and CMFs observed in cell walls.

Navon et al. (2020) introduced deposition of cellulose nanocrystals (CNCs) on a supported lipid bilayer (SLB) governed by electrostatic interactions, which provides the basis for a relevant 2D model of a PCW. The cellulose nanocrystals deposition was validated through hydrated mass, apparent thickness, and hydration. The results confirmed that adsorption strongly depends on lipid composition, cellulose nanocrystals concentration, and pH conditions. AFM topography imaging confirms the homogenous cellulose nanocrystal film with a thickness of a cellulose nanocrystal monolayer. This established a controlled formation of 2-D PCW model through decoupling cellulose assembly from bulk gel formation.

Cybulska et al. (2010) employed AFM to visualize and investigate apple parenchyma cell walls. They first investigated the effects of sample preparation techniques: parenchyma wall extraction via the buffer-phenol method (dubbed CWMR-reconstituted apple cell wall material prepared by means of the buffer-phenol method material) versus a tissue fixation by drying method (dubbed FAT-fixed apple cell wall material). AFM of the CWMR material revealed loosely organized, chaotic networks of CMFs that were long, thin, straight, and untangled. FAT material exhibited thicker and wavier fibrils, as this preparation method likely included more hemicelluloses, crosslinking proteins, and pectins (Figure 14B). In an effort to create artificial cell walls, the authors used a polysaccharide network based on BC supplemented with pectin and/or xyloglucan (Figure 14C). Quantitative assessment was provided through the AFM height profiles. The cellulose microfibrils in CWMR and FAT materials were found to be in the range from 13 nm to 22 nm in diameter, respectively. The values are in agreement with previously reported values for PCW fibrils (6–25 nm) (Thimm et al., 2000). AFM revealed that the incorporation of pectin (44%) and xyloglucan (20%) with BC (26%) produced thicker, cohesive microfibrils, which, although significantly different in morphology, most closely mimicked the natural apple parenchyma cell wall samples.

Jean and coworkers (Jean et al., 2009) (Non-Electrostatic Building of Biomimetic Cellulose-Xyloglucan Multilayers) introduced a multi -layered alternating cellulose nanocrystals and xyloglucan composite, which is analogous to the architecture of hemicellulose-cellulose microfibrils in primary cell walls. AFM topography validates that the layers do not possess rough and aggregate-prone coating, showing linear growth as the number of layers increases. This stratified architecture resembles PCW, because cellulose nanocrystal layers are well separated by thin xyloglucan spacers (Figure 14D).

**FIGURE 15:**
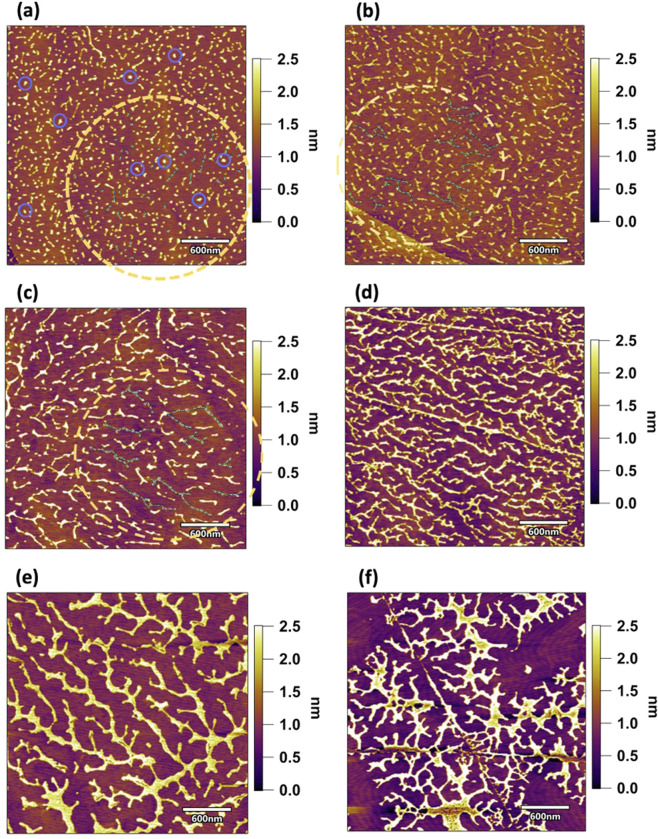
AFM topography image P1 EXT precursor (5 μg/mL) on highly oriented pyrolytic graphite (HOPG) incubated for **(a)** 1 min, **(b)** 2 min, **(c)** 4 min, **(d)** 8 min, **(e)** 10 min, and **(f)** 16 min (Ambagaspitiya et al., 2025).

AFM has been used to examine the self-assembling properties of plant cell wall extensin glycoproteins (EXTs) (Ambagaspitiya et al., 2025; Cannon et al., 2008). EXTs possess many of the properties of biomaterials discussed above: they are di- and/or tri-block copolymers, monomers generally adopt PPII helices, which give rod-like extended conformations, and they are self-assembling amphiphiles. EXTs are rich in hydroxyproline residues that subsequently become highly O-glycosylated. EXTs are composed of alternating hydrophilic peptide glycomodules and hydrophobic crosslinking modules. In the wall, EXTs play roles in controlling cell growth and division, embryo development, and are major players in wall remodeling in response to biotic and abiotic stresses. The functions of EXTs are governed by their self-assembly in the wall and subsequent covalent crosslinking. Recently, Ambagaspitiya *et al.* investigated the effects of incubation time and monomer concentration on EXT self-assembly using AFM (Ambagaspitiya et al., 2025). Varying the P1 precursor concentration from 5 to 100 μg/mL, the surface coverage increased from 12% to 95%, yielding a layer of assembled EXTs. Moreover, different incubation times at a 5 μg/mL P1 concentration reveal that longer incubation time yields elongated and branched features due to stacking and parallel interactions of P1 monomers (Figure 15). These studies provide insights into the self-assembly of EXTs, thus facilitating understanding of network formation under various experimental conditions. The knowledge of synthetic EXTs provides crucial information of complex molecular-level arrangements within plant cell walls, which in turn, can be integrated into the design of next-generation biomimetic cell wall-like polymers.

Beyond topographical imaging, distinct AFM modes such as force-distance mapping, chemical force microscopy, and tapping/phase mapping can enable image evaluation of plant and mimetic cell wall materials for quantifying stiffness, viscoelasticity, surface polarity, and interfacial adhesion. For example, AFM force spectroscopy can allow us to probe where and how adhesion interactions and stiffness vary across cell wall structures via recording force versus distance at high resolution. These data can then be transformed into force-indentation curves, followed by mathematical fitting using continuum elastic models. These elastic models were developed for a specific contact geometry between the sample surface and the AFM tip. The tailored models for different tip-sample contacts can then be used to calculate the elastic modulus of the sample. There are well-known contact models available for distinct contact behaviours on flat and stiff surfaces, such as Hertz, Johnson-Kendall-Roberts (JKR) (Johnson et al., 1971) and Derjaguin-Muller-Toporov (DMT) (Derjaguin et al., 1975). The Hertz model is applied when small deformation and negligible adhesion forces between the tip and the sample surface are assumed. However, this model does not consider potential viscoelastic behaviors of plant cell wall materials. Conversely, JKR and DMT models consider adhesion forces but are applicable to two different types of material. While the JKR model is compliant for soft materials, the DMT model is preferable for hard materials (Ramasinghe and Cimatu, 2023). Hence, the model selection is based on the nature of the sample including sample heterogeneity, hydration state, viscoelastic behavior, etc. Plant cell wall mimetic composite comprises stiff crystalline cellulose microfibriles embedded in a soft, hydrated polysaccharide matrix. Hence, careful choice of a contact mechanics model plays an important role in the accurate estimation of the Young’s modulus.

**FIGURE 16:**
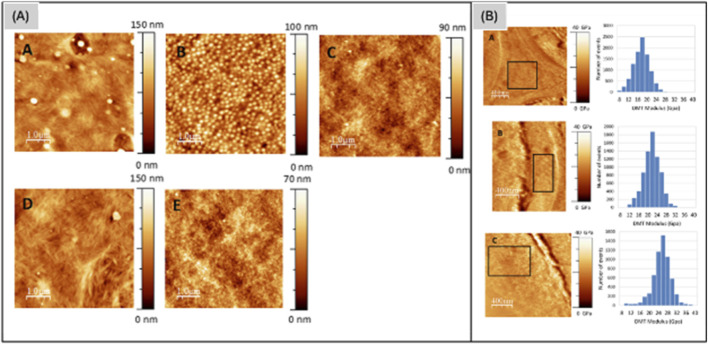
Mechanical characterization of plant cell wall-like materials **(A)** AFM images of lignified films (Muraille et al., 2017). **(B)** DMT modulus mapping of poplar fiber cell wall layers. (a) Middle lamella/primary wall, (b) S1 layer, and (c) S2 layer (Muraille et al., 2017).

Obersriebnig *et al.* (Obersriebnig et al., 2013) used the ability of AFM to map how the lamellar architecture, microfibril orientation, and matrix composition of spruce wood specimens affect their adhesion behavior with urea-formaldehyde and polyurethane. By integrating high-resolution topographical and mechanical mapping via AFM, Muraille *et al.* (Muraille et al., 2017) collected nanostructural and nanomechanical measurements of plant cell wall mimetic lignocellulosic films and natural poplar cell walls. The authors first investigated the topographical and mechanical properties. The typical layered SCW morphology was observed, consisting of sublayers of primary and secondary walls. These distinct layers show a gradient in stiffness, resulting in the highest modulus for secondary wall sublayers. In order to mimic the lignified plant cell walls, thin lignocellulosic films were fabricated from cellulose nanocrystals, hemicellulose (glucomannan or xylan), and lignin. The authors assessed both lignified and non-lignified films to confirm the emergence of a hierarchical crosslinking pattern of lignification. Non-lignified films exhibit a homogeneous topography, confirming uniform hybrid matrices, in which hemicellulose aligns along cellulose nanocrystals via hydrogen bonding. Lignified films, however, showed surface nodules. Smaller and denser nodules were observed in xylan hemicelluloses, while large nodules were observed in films containing glucomannan hemicelluloses (Figure 16A). AFM topography images revealed the biomimetic assembly of lignocellulosic films replicating the spatial heterogeneity and covalent crosslinking of natural lignification. Beyond the visual hierarchical architecture, AFM peakForce quantitative nanomechanical mapping, followed by the DMT model fitting, enabled the detection of the effect of polymer composition on stiffness. Even though non-lignified glucomannan or xylan-based systems exhibit 29 and 15 GPa, respectively, lignified films displayed 22 and 11 GPa, which closely resembled the values for the secondary wall layers in poplar fibers (Figure 16B). Overall, the successful biomimicry of nanostructural and mechanical characteristics of the poplar plant was observed in bioinspired lignocellulosic films.

In addition to hardwood SCWs (like poplar), softwood SCWs from Norway spruce were studied by Gindl et al. (2024) They performed quasi-static indentation tests with a Hysitron nanoindenter, which creates permanent deformations in wood cells, followed by light-microscopy. The permanent deformations were then fitted using the Oliver and Pharr model to measure wood stiffness. The key findings of this study showed that the elastic modulus was strongly related to microfibril orientation: increasing the microfibril angle from 0 to 50 ° decreased wall stiffness sharply. AFM nanoindentation offers superior spatial, chemical, and environmental resolution. For instance Wu et al. (2010) investigated the nanoindentation of native plant cell walls of intact plant tissues of cotton (*Gossypium herbaceu*) stalk, soybean (*Glycine max*) stalk, cassava (*Manihot esculenta*) stalk, rice (*Oryza sativa*) straw, and wheat (*Triticum aestivum*) straw. The elastic modulus of these crop stalks is found in the range of ∼ 15–30 GPa, the differences observed based on the cell type, tissue organization, and cell wall composition. Overall, this work established that nano-indentation as an effective approach to quantitatively link the SCW architecture and composition to the mechanical properties.

In summary, AFM has emerged as a powerful platform for resolving nanoscale morphology and nanomechanics of plant cell wall mimetic polymers, enabling direct visualization of fibril architectures, hydration dynamics, etc. These insights gain particular attention in prospective biomimetic materials. AFM can serve as a powerful platform to investigate the self-assembly architectures in plant cell walls, such as the self-assembly polymerization of extensin, which has recently garnered significant attention (Mishler-Elmore et al., 2021). By leveraging AFM to dissect these nanoscale assembly processes, researchers can begin to translate extensin self-assembly principles into the design of next-generation biomimetic materials, with tunable architecture, stiffness, and intrinsic reinforcement capabilities.

### Structural biomimicry perspectives

Plant cell wall mimicry depends on hierarchical polymer organization rather than composition alone. Hence, NMR, SFG, and AFM provide complementary molecular, mesoscale, and nanoscale information, which are critical to evaluate plant cell wall mimicry. Even though ssNMR and SFG can potentially detect chemical identity, NMR can analyze intact or minimally perturbed cell wall materials and distinguish polymer conformations through bulk composition, while SFG is capable of selectively detecting ordered, non-centrosymmetric molecular assemblies. NMR has methodological constraints as it requires isotopic enrichment, specialized pulse sequences, long acquisition times, high-field instrumentation, and careful spectral interpretation. On the other hand, SFG is especially useful for evaluating plant cell wall mimicry as this is sensitive not only to crystalline cellulose content but also to microfibril alignment and packing in intact plant cell walls. However, SFG also has limitations as it detects non-centrosymmetric structures; the SFG signal is often weak or absent due to prevalent disorder, centrosymmetric cancellation, random orientation, and unfavorable sample geometry rather than true absence of cellulose. AFM is capable of probing polymer morphology, surface assembly, and network formation in real space. This can quantify height, length, roughness, branching, surface coverage, modulus, and adhesion. Hence, used together, these techniques allow researchers to determine whether an engineered system reproduces the key features of plant cell walls.

## Insights

Biomimicry aims to emulate the structural sophistication, hierarchical organization, and dynamic responsiveness seen in natural systems. Biomimetic polymers have always fascinated researchers, as they lie uniquely in the intersection of biological function and synthetic material chemistry and biology. One of the challenges in the generation and fabrication of biomimetic materials is their increasing complexity, including sequence-specific structures or polymer hybrids that can respond to various stimuli similarly to how natural systems typically respond, and a need for characterization techniques that enable precise comparisons of results between native and synthetic biopolymers. The field of biomimetic polymers is restricted not only by our synthetic capabilities but also by our ability to measure and understand the structures and properties we generate. In this regard, characterization techniques that probe bulk and microscopic properties are not merely auxiliary tools but foundational elements enabling the design of next-generation biomimetic polymers and materials.

In this review, we highlight the crucial role of characterization techniques as indispensable tools in resolving hierarchical structures from the nanoscale to the mesoscale. Techniques such as SEM, XRD, and FTIR can elucidate, with a high degree of resolution, the multiscale architectures and identify emergent properties arising from these assemblies. These tools provide valuable insights into molecular design choices that could propagate simple structures, such as cotton fibers (made of cellulose) into higher-order assemblies (such as cell wall mimics). These enable researchers to fine-tune materials with desired properties suitable for a particular intended application.

Equally important are spectroscopic and chemical analysis methods that can shed light on the stability and composition of biomimetic polymers. DSC and DLS offer insights about thermal stability and particle size distribution, respectively. Taken together, they are important techniques that capture transient states observed the microscopic level and bridge them to the measured macroscopic-level properties. NMR, SFG, and AFM can be used to verify the presence of functionalities, monitor conformational changes, and identify noncovalent forces that stabilize the biomimetic motifs and architectures. Despite rapid advances in biomimetic materials research, true plant-based biomimetics remain underrepresented in the literature. In contrast to the extensive studies on animal-inspired systems such as adhesive proteins, structural proteins, and extracellular matrices, plant-based systems, like the structural glycoproteins, are sparsely reported. This reflects the inherent complexity and heterogeneity of plant glycoproteins, posing challenges for precise molecular and analytical characterization.

Plant-based structural glycoproteins–extensins and arabinogalactan proteins–offer an inherently strategic opportunity for new and sustainable inspirations of next-generation biomimetic materials. In addition to their abundance and renewability, they can also exhibit tunable properties and can self-assemble remarkably. The creation of true biomimetics derived from these structural glycoproteins requires the precise understanding of molecular-level mechanisms that govern their self-assembly. Our current work introduces a synthetic biology platform for the construction of custom glycoproteins that recapitulate the essential features of these glycoproteins. We are currently engineering synthetic analogs that harness the unique yet predictable, glycosylation patterns used by plants to access molecular configurations that are not accessible by conventional synthetic/chemical methods. By studying the glycan composition, spacing, and cross-linking motifs that dictate the macromolecular structure and architectures of these glycoproteins, we can translate and extend this to design novel plant-based biomimetics. Our work aims to fill a long-standing gap in the search for plant-based biomimetics to design sustainable materials. This review examines and intends to emphasize plant biological systems and models–long overshadowed by animal models in the design of biomimetic materials–may offer a versatile, environmentally-friendly, and more sustainable blueprint for the fabrication of the next-generation of high-performance materials.

## References

[B1] Agnarsson I. Kuntner M. Blackledge T. A. (2010). Bioprospecting finds the toughest biological material: extraordinary silk from a giant riverine orb spider. PLoS One 5 (9), e11234. 10.1371/journal.pone.0011234 20856804 PMC2939878

[B2] Ambagaspitiya T. D. Ramasinghe L. P. Sukul A. Regunton A. K. Cimatu K. L. A. Held M. A. (2025). Assessing the self-assembly of native P1 extensin glycoproteins on highly oriented pyrolytic graphite (HOPG) using atomic force microscopy (AFM). Langmuir 42 (1), 118–128. 10.1021/acs.langmuir.5c03261 41294111

[B3] Aoyagi S. Izumi H. Fukuda M. (2008). Biodegradable polymer needle with various tip angles and consideration on insertion mechanism of mosquito's proboscis. Sensors Actuators A Phys. 143 (1), 20–28. 10.1016/j.sna.2007.06.007

[B4] Balu R. Dutta N. K. Dutta A. K. Choudhury N. R. (2021). Resilin-mimetics as a smart biomaterial platform for biomedical applications. Nat. Commun. 12 (1), 149. 10.1038/s41467-020-20375-x 33420053 PMC7794388

[B5] Bandiera A. Colomina - Alfaro L. Sist P. Gomez d’Ayala G. Zuppardi F. Cerruti P. (2023). Physicochemical characterization of a biomimetic, elastin-inspired polypeptide with enhanced thermoresponsive properties and improved cell adhesion. Biomacromolecules 24 (11), 5277–5289. 10.1021/acs.biomac.3c00782 37890135 PMC10647011

[B6] Barnette A. L. Bradley L. C. Veres B. D. Schreiner E. P. Park Y. B. Park J. (2011). Selective detection of crystalline cellulose in plant cell walls with sum-frequency-generation (SFG) vibration spectroscopy. Biomacromolecules 12 (7), 2434–2439. 10.1021/bm200518n 21615075

[B7] Beynus J. (1997). Biomimicry: Innovation Inspired by Nature. New York, NY: Perennial.

[B8] Bootten T. J. Harris P. J. Melton L. D. Newman R. H. (2009). Solid-state 13C NMR study of a composite of tobacco xyloglucan and Gluconacetobacter xylinus cellulose: molecular interactions between the component polysaccharides. Biomacromolecules 10 (11), 2961–2967. 10.1021/bm900762m 19817435

[B9] Bowen C. H. Dai B. Sargent C. J. Bai W. Ladiwala P. Feng H. (2018). Recombinant spidroins fully replicate primary mechanical properties of natural spider silk. Biomacromolecules 19 (9), 3853–3860. 10.1021/acs.biomac.8b00980 30080972

[B10] Brunel L. G. Long C. M. Christakopoulos F. Cai B. Johansson P. K. Singhal D. (2025). Interpenetrating networks of fibrillar and amorphous collagen promote cell spreading and hydrogel stability. Acta Biomater. 193, 128–142. 10.1016/j.actbio.2025.01.009 39798635 PMC11908676

[B11] Cannon M. C. Terneus K. Hall Q. Tan L. Wang Y. Wegenhart B. L. (2008). Self-assembly of the plant cell wall requires an extensin scaffold. Proc. Natl. Acad. Sci. 105 (6), 2226–2231. 10.1073/pnas.0711980105 18256186 PMC2538902

[B12] Castillo-Paz A. M. García-Vazquez K. V. Cañon-Davila D. F. Hernandez-Landaverde M. A. Chan-Chan L. H. Ramírez-Bon R. (2024). *In vitro* immersion study and characterization of biomimetic bovine hydroxyapatite scaffolds: influence of calcination temperature (600 and 1000 °C) on apatite formation. Ceram. Int. 50 (15), 26949–26962. 10.1016/j.ceramint.2024.04.426

[B13] Chan N. J. Lentz S. Gurr P. A. Scheibel T. Qiao G. G. (2022). Mimicry of silk utilizing synthetic polypeptides. Prog. Polym. Sci. 130, 101557. 10.1016/j.progpolymsci.2022.101557

[B14] Chilkoti A. Christensen T. MacKay J. A. (2006). Stimulus responsive elastin biopolymers: applications in medicine and biotechnology. Curr. Opin. Chem. Biol. 10 (6), 652–657. 10.1016/j.cbpa.2006.10.010 17055770 PMC3732176

[B15] Choi J.-W. Choi S.-H. Won J.-I. (2021). Self-assembly behavior of elastin-like polypeptide diblock copolymers containing a charged moiety. Biomacromolecules 22 (6), 2604–2613. 10.1021/acs.biomac.1c00322 34038105

[B16] Ciulla M. G. Civera M. Sattin S. Kumar K. (2023). Nature-inspired and medicinally relevant short peptides. Explor. Drug Sci. 1 (3), 140–171. 10.37349/eds.2023.00011

[B17] Cuéllar-Cruz M. Pérez K. S. Mendoza M. E. Moreno A. (2020). Biocrystals in plants: a short review on biomineralization processes and the role of phototropins into the uptake of calcium. Crystals 10 (7), 591. 10.3390/cryst10070591

[B18] Cybulska J. Konstankiewicz K. Zdunek A. Skrzypiec K. (2010). Nanostructure of natural and model cell wall materials. Int. Agrophysics 24 (2), 107–114.

[B19] Dai M. Georgilis E. Goudounet G. Garbay B. Pille J. van Hest J. C. M. (2021). Refining the design of diblock elastin-like polypeptides for self-assembly into nanoparticles. Polymers 13 (9), 1470. 10.3390/polym13091470 34062852 PMC8125372

[B20] Dandurand J. Samouillan V. Lacabanne C. Pepe A. Bochicchio B. (2018). Phase behavior and chain dynamics of elastin-like peptides versus amino acid sequences. J. Therm. Analysis Calorim. 131 (2), 1323–1332. 10.1007/s10973-017-6633-5

[B21] Debnath A. Mitra S. Ghosh S. Sen R. (2024). Understanding microbial biomineralization at the molecular level: recent advances. World J. Microbiol. Biotechnol. 40 (10), 320. 10.1007/s11274-024-04132-6 39279013

[B22] Derjaguin B. V. Muller V. M. Toporov Y. P. (1975). Effect of contact deformations on the adhesion of particles. J. Colloid Interface Science 53 (2), 314–326. 10.1016/0021-9797(75)90018-1

[B23] Dodda J. M. Deshmukh K. Bezuidenhout D. Yeh Y.-C. (2023). “Hydrogels: definition, history, classifications, formation, constitutive characteristics, and applications,” in Multicomponent Hydrogels: Smart Materials for Biomedical Applications. Editors Dodda J. M. Deshmukh K. Bezuidenhout D. (Cambridge, United Kingdom: The Royal Society of Chemistry), 0.

[B24] Doyle A. D. Wang F. W. Matsumoto K. Yamada K. M. (2009). One-dimensional topography underlies three-dimensional fibrillar cell migration. J. Cell Biol. 184 (4), 481–490. 10.1083/jcb.200810041 19221195 PMC2654121

[B25] Dupree R. Simmons T. J. Mortimer J. C. Patel D. Iuga D. Brown S. P. (2015). Probing the molecular architecture of Arabidopsis thaliana secondary cell walls using two-and three-dimensional 13C solid state nuclear magnetic resonance spectroscopy. Biochemistry 54 (14), 2335–2345. 10.1021/bi501552k 25739924

[B26] Fatimah S. Ragadhita R. Al Husaeni D. F. Nandiyanto A. B. D. (2022). How to calculate crystallite size from x-ray diffraction (XRD) using scherrer method. ASEAN J. Sci. Eng. 2 (1), 65–76. 10.17509/ajse.v2i1.37647

[B27] Foston M. (2014). Advances in solid-state NMR of cellulose. Curr. Opinion Biotechnology 27, 176–184. 10.1016/j.copbio.2014.02.002 24590189

[B28] Galeano S. García‐Lorenzo M. L. (2014). Bone mineral change during experimental calcination: an X‐ray diffraction study. J. Forensic Sciences 59 (6), 1602–1606. 10.1111/1556-4029.12525 24962811

[B29] Gindl W. Gupta H. Schöberl T. Lichtenegger H. Fratzl P. (2004). Mechanical properties of spruce wood cell walls by nanoindentation. Appl. Phys. A 79 (8), 2069–2073. 10.1007/s00339-004-2864-y

[B30] Gonzalez-Obeso C. Rodriguez-Cabello J. C. Kaplan D. L. (2022). Fast and reversible crosslinking of a silk elastin-like polymer. Acta Biomater. 141, 14–23. 10.1016/j.actbio.2021.12.031 34971785 PMC8898266

[B31] Graham J. J. Subramani S. V. Yang X. Russell T. M. Zhang F. Keten S. (2025). Charting the envelope of mechanical properties of synthetic silk fibers through predictive modeling of the drawing process. Sci. Adv. 11 (10), eadr3833. 10.1126/sciadv.adr3833 40053589 PMC11887809

[B32] Guo Y. Liu S. Jing D. Liu N. Luo X. (2023). The construction of elastin-like polypeptides and their applications in drug delivery system and tissue repair. J. Nanobiotechnology 21 (1), 418. 10.1186/s12951-023-02184-8 37951928 PMC10638729

[B33] Hashempur M. H. Radmanesh A. Karami F. Zomorodian K. Amirzadeh N. Shenavari S. (2025). Fabrication of a sponge-like protein based hydrogel incorporating fluconazole against candida species as a potential treatment for vulvovaginal candidiasis infection. Sci. Rep. 15 (1), 24364. 10.1038/s41598-025-09457-2 40628878 PMC12238399

[B34] Hill S. J. Franich R. A. Callaghan P. T. Newman R. H. (2009). Nature’s nanocomposites: a new look at molecular architecture in wood cell walls. NZJ Sci. 39, 251–257.

[B35] Huang D. Li Z. Li G. Zhou F. Wang G. Ren X. (2025). Biomimetic structural design in 3D-printed scaffolds for bone tissue engineering. Mater. Today Bio 32, 101664. 10.1016/j.mtbio.2025.101664 PMC11979411 40206144

[B36] Huang X. Yu W. Gu W. Liang S. Zhou L. Zhang L. (2025). Mimicking natural biomineralization enabling biodegradable and highly lipophobic alginate hydrogels. Carbohydr. Polym. 357, 123438. 10.1016/j.carbpol.2025.123438 40158976

[B37] Ifergan-Azriel L. Bar-Am O. Saar G. Cohen T. Loebel C. Burdick J. A. (2025). Interpenetrating polymer network hydrogel composition alters encapsulated MSC spreading and *in vivo* degradation behavior. ACS Biomaterials Sci. and Eng. 11 (9), 5586–5599. 10.1021/acsbiomaterials.5c00980 PMC12421499 40757659

[B38] Ismail A. A. M. Ghanem L. G. Akar A. A. Khedr G. E. Ramadan M. Shaheen B. S. (2023). Novel self-regenerative and non-flammable high-performance hydrogel electrolytes with anti-freeze properties and intrinsic redox activity for energy storage applications. J. Mater. Chem. A 11 (30), 16009–16018. 10.1039/d3ta02499g

[B39] Jamburidze A. De Corato M. Huerre A. Pommella A. Garbin V. (2017). High-frequency linear rheology of hydrogels probed by ultrasound-driven microbubble dynamics. Soft Matter 13 (21), 3946–3953. 10.1039/c6sm02810a 28504278

[B40] Jean B. Heux L. Dubreuil F. Chambat G. Cousin F. (2009). Non-electrostatic building of biomimetic cellulose− xyloglucan multilayers. Langmuir 25 (7), 3920–3923. 10.1021/la802801q 18986190

[B41] Jeong J. Kim J. H. Shim J. H. Hwang N. S. Heo C. Y. (2019). Bioactive calcium phosphate materials and applications in bone regeneration. Biomaterials Research 23 (1), 4. 10.1186/s40824-018-0149-3 30675377 PMC6332599

[B42] Johnson K. L. Kendall K. Roberts A. (1971). Surface energy and the contact of elastic solids. Proc. Royal Society Lond. A Mathematical Physical Sciences 324 (1558), 301–313. 10.1098/rspa.1971.0141

[B43] Kadi F. Dini G. Poursamar S. A. Ejeian F. (2024). Fabrication and characterization of 3D-printed composite scaffolds of coral-derived hydroxyapatite nanoparticles/polycaprolactone/gelatin carrying doxorubicin for bone tissue engineering. J. Mater. Sci. Mater. Med. 35 (1), 7. 10.1007/s10856-024-06779-x 38285297 PMC10824813

[B44] Kang X. Kirui A. Dickwella Widanage M. C. Mentink-Vigier F. Cosgrove D. J. Wang T. (2019). Lignin-polysaccharide interactions in plant secondary cell walls revealed by solid-state NMR. Nat. Communications 10 (1), 347. 10.1038/s41467-018-08252-0 30664653 PMC6341099

[B45] Kim W. Chaikof E. L. (2010). Recombinant elastin-mimetic biomaterials: emerging applications in medicine. Adv. Drug Deliv. Rev. 62 (15), 1468–1478. 10.1016/j.addr.2010.04.007 20441783 PMC2937194

[B46] Kongsri S. Janpradit K. Buapa K. Techawongstien S. Chanthai S. (2013). Nanocrystalline hydroxyapatite from fish scale waste: preparation, characterization and application for selenium adsorption in aqueous solution. Chem. Engineering Journal 215, 522–532. 10.1016/j.cej.2012.11.054

[B47] Koshani R. Pitcher M. L. Yu J. Mahajan C. L. Kim S. H. Sheikhi A. (2025). Plant cell wall-like soft materials: micro-and nanoengineering, properties, and applications. Nano-Micro Lett. 17 (1), 103. 10.1007/s40820-024-01569-0 PMC11711842 39777633

[B48] Koshy P. Abdullah H. Idris M. Lee T. (2019). Syntheses of hydroxyapatite from natural sources. Heliyon 5 (5). 10.1016/j.heliyon.2019.e01588 PMC6507053 31080905

[B49] Kozioł A. Cybulska J. Pieczywek P. M. Zdunek A. (2015). Evaluation of structure and assembly of xyloglucan from tamarind seed (Tamarindus indica L.) with atomic force microscopy. Food Biophys. 10 (4), 396–402. 10.1007/s11483-015-9395-2 26523128 PMC4623076

[B50] Le T. D. H. Van Le H. Nguyen G. T. Le A. T. (2025). Novel chitosan/starch scaffold supplemented with hydroxyapatite nanoparticles enhanced mineralization for bone regeneration: processing and characterization. Mater. Res. Express 12 (9), 095401. 10.1088/2053-1591/ae0417

[B51] Lee C. M. Kafle K. Park Y. B. Kim S. H. (2014). Probing crystal structure and mesoscale assembly of cellulose microfibrils in plant cell walls, tunicate tests, and bacterial films using vibrational sum frequency generation (SFG) spectroscopy. Phys. Chem. Chem. Phys. 16 (22), 10844–10853. 10.1039/c4cp00515e 24760365

[B52] Lee J. Chaves A. M. Choi J. Roberts A. W. Kim S. H. (2023). Sum frequency generation (SFG) microscopy analysis of cellulose microfibrils in Physcomitrium patens gametophore leaf. Cellulose 30 (13), 8395–8404. 10.1007/s10570-023-05355-w

[B53] Leng Y. Abdullah A. Wendt M. K. Calve S. (2019). Hyaluronic acid, CD44 and RHAMM regulate myoblast behavior during embryogenesis. Matrix Biol. 78-79, 236–254. 10.1016/j.matbio.2018.08.008 30130585 PMC6379145

[B54] Li L. Teller S. Clifton R. J. Jia X. Kiick K. L. (2011). Tunable mechanical stability and deformation response of a Resilin-Based elastomer. Biomacromolecules 12 (6), 2302–2310. 10.1021/bm200373p 21553895 PMC3139215

[B55] Li G. Gao F. Yang D. Lin L. Yu W. Tang J. (2024). ECM-mimicking composite hydrogel for accelerated vascularized bone regeneration. Bioact. Mater. 42, 241–256. 10.1016/j.bioactmat.2024.08.035 39285909 PMC11404060

[B56] Li A. A.-O. Putra K. B. Chen L. Montgomery J. S. Shih A. (2045). Mosquito proboscis-inspired Needle Insertion to Reduce Tissue Deformation and Organ Displacement–2322. (Electronic)). 10.1038/s41598-020-68596-w PMC7376018 32699296

[B57] Lin J. Cai Y. Wang X. Ding B. Yu J. Wang M. (2011). Fabrication of biomimetic superhydrophobic surfaces inspired by lotus leaf and silver ragwort leaf. Nanoscale 3 (3), 1258–1262. 10.1039/c0nr00812e 21270991

[B58] Liu Z. Ling S. D. Liang K. Chen Y. Niu Y. Sun L. (2024). Viscoelasticity of ECM and cells—origin, measurement and correlation. Mechanobiol. Med. 2 (4), 100082. 10.1016/j.mbm.2024.100082 40395221 PMC12082326

[B59] Liu X. Hu H. Ma J. Wang B. (2025). Mineralized cellulose nanofibers reinforced bioactive hydrogel remodels the osteogenic and angiogenic microenvironment for enhancing bone regeneration. Carbohydr. Polym. 357, 123480. 10.1016/j.carbpol.2025.123480 40159001

[B60] López Barreiro D. Minten I. J. Thies J. C. Sagt C. M. J. (2023). Structure–property relationships of elastin-like polypeptides: a review of experimental and computational studies. ACS Biomaterials Sci. and Eng. 9 (7), 3796–3809. 10.1021/acsbiomaterials.1c00145 34251181

[B61] López Barreiro D. Houben K. Schouten O. Koenderink G. H. Thies J. C. Sagt C. M. J. (2025). Order–Disorder balance in silk-elastin-like polypeptides determines their self-assembly into hydrogel networks. ACS Appl. Mater. and Interfaces 17 (1), 650–662. 10.1021/acsami.4c17903 39681513 PMC11783522

[B62] Lou J. Mooney D. J. (2022). Chemical strategies to engineer hydrogels for cell culture. Nat. Rev. Chem. 6 (10), 726–744. 10.1038/s41570-022-00420-7 37117490

[B63] Lou J. Stowers R. Nam S. Xia Y. Chaudhuri O. (2018). Stress relaxing hyaluronic acid-collagen hydrogels promote cell spreading, fiber remodeling, and focal adhesion formation in 3D cell culture. Biomaterials 154, 213–222. 10.1016/j.biomaterials.2017.11.004 29132046

[B64] Lowenstam H. A. Weiner S. (1989). On Biomineralization. Oxford University Press.

[B65] Luo M. Li Z. Su M. Gadd G. M. Yin Z. Benton M. J. (2023). Fungal-induced fossil biomineralization. Curr. Biol. 33 (12), 2417. 10.1016/j.cub.2023.04.067 37230078

[B66] Makarem M. Lee C. M. Kafle K. Huang S. Chae I. Yang H. (2019). Probing cellulose structures with vibrational spectroscopy. Cellulose 26 (1), 35–79. 10.1007/s10570-018-2199-z

[B67] McGann C. L. Akins R. E. Kiick K. L. (2016). Resilin-PEG Hybrid Hydrogels Yield Degradable Elastomeric Scaffolds with Heterogeneous Microstructure. Biomacromolecules 17 (1), 128–140. 10.1021/acs.biomac.5b01255 26646060 PMC4850080

[B68] Mei D. Li N. Yan K. Wang J. Li X. You R. (2025). Natural silk nanofibril-directed mineralization for biomimetic scaffolds. Biomacromolecules 26 (9), 6070–6081. 10.1021/acs.biomac.5c01006 40814200

[B69] Meng L. Xie F. Zhang B. Wang D. K. Yu L. (2019). Natural biopolymer alloys with superior mechanical properties. ACS Sustain. Chem. and Eng. 7 (2), 2792–2802. 10.1021/acssuschemeng.8b06009

[B70] Mi J. Zhou Y. Ma S. Zhou X. Xu S. Yang Y. (2023). High-strength and ultra-tough whole spider silk fibers spun from transgenic silkworms. Matter 6 (10), 3661–3683. 10.1016/j.matt.2023.08.013

[B71] Milne C. Song R. Johnson M. Zhao C. Santoro Ferrer F. A S. (2024). Dual-modified hyaluronic acid for tunable double cross-linked hydrogel adhesives. Biomacromolecules 25 (4), 2645–2655. 10.1021/acs.biomac.4c00194 38456398 PMC11005013

[B72] Mishler-Elmore J. W. Zhou Y. Sukul A. Oblak M. Tan L. Faik A. (2021). Extensins: self-assembly, crosslinking, and the role of peroxidases. Front. Plant Sci. 12, 664738. 10.3389/fpls.2021.664738 34054905 PMC8160292

[B73] Mohammadi P. Aranko A. S. Landowski C. P. Ikkala O. Jaudzems K. Wagermaier W. (2019). Biomimetic composites with enhanced toughening using silk-inspired triblock proteins and aligned nanocellulose reinforcements. Sci. Adv. 5 (9), eaaw2541. 10.1126/sciadv.aaw2541 31548982 PMC6744269

[B74] Montalbano G. Toumpaniari S. Popov A. Duan P. Chen J. Dalgarno K. (2018). Synthesis of bioinspired collagen/alginate/fibrin based hydrogels for soft tissue engineering. Mater. Sci. Eng. C 91, 236–246. 10.1016/j.msec.2018.04.101 30033251

[B75] Muir V. G. Burdick J. A. (2021). Chemically modified biopolymers for the formation of biomedical hydrogels. Chem. Rev. 121 (18), 10908–10949. 10.1021/acs.chemrev.0c00923 33356174 PMC8943712

[B76] Muiznieks L. D. Keeley F. W. (2013). Molecular assembly and mechanical properties of the extracellular matrix: a fibrous protein perspective. Biochimica Biophysica Acta (BBA) - Mol. Basis Dis. 1832 (7), 866–875. 10.1016/j.bbadis.2012.11.022 23220448

[B77] Mundekkad D. Mallya A. R. (2025). Biomimicry at the nanoscale - a review of nanomaterials inspired by nature. Nano Trends 10, 100119. 10.1016/j.nwnano.2025.100119

[B78] Muraille L. Aguié-Béghin V. Chabbert B. Molinari M. (2017). Bioinspired lignocellulosic films to understand the mechanical properties of lignified plant cell walls at nanoscale. Sci. Reports 7 (1), 44065. 10.1038/srep44065 28276462 PMC5343475

[B79] Nam S. Mooney D. (2021). Polymeric tissue adhesives. Chem. Rev. 121 (18), 11336–11384. 10.1021/acs.chemrev.0c00798 33507740

[B80] Navon Y. Jean B. Coche-Guérente L. Dahlem F. Bernheim-Groswasser A. Heux L. (2020). Deposition of cellulose nanocrystals onto supported lipid membranes. Langmuir 36 (6), 1474–1483. 10.1021/acs.langmuir.9b02888 31904979

[B81] Neacsu I. A. Serban A. P. Nicoara A. I. Trusca R. Ene V. L. Iordache F. (2020). Biomimetic composite scaffold based on naturally derived biomaterials. Polymers 12 (5), 1161. 10.3390/polym12051161 32438578 PMC7284724

[B82] Norioka C. Inamoto Y. Hajime C. Kawamura A. Miyata T. (2021). A universal method to easily design tough and stretchable hydrogels. NPG Asia Mater. 13 (1), 34. 10.1038/s41427-021-00302-2

[B83] Obersriebnig M. Konnerth J. Gindl-Altmutter W. (2013). Evaluating fundamental position-dependent differences in wood cell wall adhesion using nanoindentation. Int. Journal Adhesion Adhesives 40, 129–134. 10.1016/j.ijadhadh.2012.08.011 27570321 PMC4986323

[B84] Park Y. B. Lee C. M. Koo B.-W. Park S. Cosgrove D. J. Kim S. H. (2013). Monitoring meso-scale ordering of cellulose in intact plant cell walls using sum frequency generation spectroscopy. Plant Physiol. 163 (2), 907–913. 10.1104/pp.113.225235 23995148 PMC3793067

[B85] Park Y. B. Lee C. M. Kafle K. Park S. Cosgrove D. J. Kim S. H. (2014). Effects of plant cell wall matrix polysaccharides on bacterial cellulose structure studied with vibrational sum frequency generation spectroscopy and x-ray diffraction. Biomacromolecules 15 (7), 2718–2724. 10.1021/bm500567v 24846814

[B86] Rahman M. S. Shon A. Joseph R. Pavlov A. Stefanov A. Namkoong M. (2025). Soft, stretchable conductive hydrogels for high-performance electronic implants. Sci. Adv. 11 (12), eads4415. 10.1126/sciadv.ads4415 40117365 PMC11927610

[B87] Ramasinghe L. P. Cimatu K. L. A. (2023). Quantifying elasticity maps of methacrylate-based copolymers using atomic force microscopy. MRS Commun. 13, 1–10. 10.1557/s43579-023-00459-7

[B88] Ramezaniaghdam M. Nahdi N. D. Reski R. (2022). Recombinant spider silk: promises and bottlenecks. Front. Bioeng. Biotechnol. 10, 835637–2022. 10.3389/fbioe.2022.835637 35350182 PMC8957953

[B89] Rodríguez-Cabello J. C. González de Torre I. Ibañez-Fonseca A. Alonso M. (2018). Bioactive scaffolds based on elastin-like materials for wound healing. Adv. Drug Deliv. Rev. 129, 118–133. 10.1016/j.addr.2018.03.003 29551651

[B90] Ronholm J. Schumann D. Sapers H. M. Izawa M. Applin D. Berg B. (2014). A mineralogical characterization of biogenic calcium carbonates precipitated by heterotrophic bacteria isolated from cryophilic polar regions. Geobiology 12 (6), 542–556. 10.1111/gbi.12102 25256888

[B92] Shah R. Huang S. Pingali S. V. Sawada D. Pu Y. Rodriguez Jr M. (2018). Hemicellulose–cellulose composites reveal differences in cellulose organization after dilute acid pretreatment. Biomacromolecules 20 (2), 893–903. 10.1021/acs.biomac.8b01511 30554514

[B93] Shao C. Zhang Z. Jin W. Zhang Z. Jin B. Jiang S. (2023). Oriented crystallization of hydroxyapatite in self-assembled peptide fibrils as a bonelike material. ACS Biomaterials Sci. and Eng. 9 (4), 1808–1814. 10.1021/acsbiomaterials.1c00713 34855358

[B94] Skardal A. Atala A. (2015). Biomaterials for integration with 3-D bioprinting. Ann. Biomed. Eng. 43 (3), 730–746. 10.1007/s10439-014-1207-1 25476164

[B95] Snyder P. J. Allard V. Bhagia S. Farahi R. H. Lereu A. L. Backlund M. P. (2025). Hemicellulose modulates nanoscale lignin architecture in synthetic plant cell walls. ACS Nano 19 (47), 40364–40382. 10.1021/acsnano.5c09006 41248213

[B96] Song J. Gerecht S. (2023). Hydrogels to recapture extracellular matrix cues that regulate vascularization. Arterioscler. Thromb. Vasc. Biol. 43 (8), e291–e302. 10.1161/atvbaha.122.318235 37317849

[B97] Su R. S. C. Kim Y. Liu J. C. (2014). Resilin: protein-based elastomeric biomaterials. Acta Biomater. 10 (4), 1601–1611. 10.1016/j.actbio.2013.06.038 23831198

[B98] Sumiyoshi S. Suyama K. Tanaka N. Andoh T. Nagata A. Tomohara K. (2022). Development of truncated elastin-like peptide analogues with improved temperature-response and self-assembling properties. Sci. Rep. 12 (1), 19414. 10.1038/s41598-022-23940-0 36371418 PMC9653453

[B99] Sun B. (2021). The mechanics of fibrillar collagen extracellular matrix. Lid. - 100515 [pii] Lid. 2, 2666–3864. (Electronic)). 10.1016/j.xcrp.2021.100515 PMC8415638 34485951

[B100] Suneetha M. Kim H. Han S. S. (2024). Bone-like apatite formation in biocompatible phosphate-crosslinked bacterial cellulose-based hydrogels for bone tissue engineering applications. Int. J. Biol. Macromol. 256, 128364. 10.1016/j.ijbiomac.2023.128364 38000603

[B101] Tan C. Sun Z. Hong Y. Li Y. Chen X. Zhang X. (2013). Reverse-biomineralization assembly of acid-sensitive biomimetic fibers for hard tissue engineering and drug delivery. J. Mater. Chem. B 1 (30), 3694–3704. 10.1039/c3tb20274g 32261267

[B102] Tang R. Suzuki M. (2023). Special issue on biomineralization: from principles to practices. ACS Biomaterials Sci. and Eng. 9 (4), 1730–1732. 10.1021/acsbiomaterials.3c00258 37032636

[B103] Thimm J. C. Burritt D. J. Ducker W. A. Melton L. D. (2000). Celery (Apium graveolens L.) parenchyma cell walls examined by atomic force microscopy: effect of dehydration on cellulose microfibrils. Planta 212 (1), 25–32. 10.1007/s004250000359 11219580

[B104] Trancik J. E. Czernuszka J. T. Bell F. I. Viney C. (2006). Nanostructural features of a spider dragline silk as revealed by electron and X-ray diffraction studies. Polymer 47 (15), 5633–5642. 10.1016/j.polymer.2005.01.110

[B105] Urry D. W. (1997). Physical chemistry of biological free energy transduction as demonstrated by elastic protein-based polymers. The J. Phys. Chem. B 101 (51), 11007–11028. 10.1021/jp972167t

[B106] van Beek J. D. Hess S. Vollrath F. Meier B. H. (2002). The molecular structure of spider dragline silk: folding and orientation of the protein backbone. Proc. Natl. Acad. Sci. U. S. A. 99 (16), 10266–10271. 10.1073/pnas.152162299 12149440 PMC124902

[B107] Wang R. Ozsvar J. Yeo G. C. Weiss A. S. (2019). Hierarchical assembly of elastin materials. Curr. Opin. Chem. Eng. 24, 54–60. 10.1016/j.coche.2019.01.004

[B108] Wang W. Liu X. Zheng X. Jin H. J. Li X. (2020). Biomineralization: an opportunity and challenge of nanoparticle drug delivery systems for cancer therapy. Adv. Healthc. Mater. 9 (22), 2001117. 10.1002/adhm.202001117 33043640

[B109] Wang, H. Bai T. Yusoff M. Khairuddin NAAC A'srai A. I. M. Razali M. H. (2025). Development of nano hydroxyapatite loaded gellan gum nanocomposite scaffold for the regeneration of bone tissue affected by osteosarcoma. Results Chem. 15, 102208. 10.1016/j.rechem.2025.102208

[B110] Wang, Y. Zhang R. Qiao Z. Dou B. Xu H. Meng F. (2025). Polyacrylamide-based hydrogel with biocompatibility and tunable stiffness for three-dimensional cell culture. ACS Appl. Bio Mater. 8 (3), 2356–2364. 10.1021/acsabm.4c01846 39949138

[B111] Wei S. Cui H. Jiang Z. Liu H. He H. Fang N. (2015). Biomineralization processes of calcite induced by bacteria isolated from marine sediments. Braz. J. Microbiol. 46 (2), 455–464. 10.1590/S1517-838246220140533 26273260 PMC4507537

[B112] Wu Y. Wang S. Zhou D. Xing C. Zhang Y. Cai Z. (2010). Evaluation of elastic modulus and hardness of crop stalks cell walls by nano-indentation. Bioresour. Technol. 101 (8), 2867–2871. 10.1016/j.biortech.2009.10.074 19954968

[B113] Xu D. Asai D. Chilkoti A. Craig S. L. (2012). Rheological properties of cysteine-containing elastin-like polypeptide solutions and hydrogels. Biomacromolecules 13 (8), 2315–2321. 10.1021/bm300760s 22789001 PMC3418688

[B114] Xu Z. Chen H. Yang H.-B. Yao X. Qin H. Cong H.-P. (2025). Hierarchically aligned heterogeneous core-sheath hydrogels. Nat. Commun. 16 (1), 400. 10.1038/s41467-024-55677-x 39755695 PMC11700098

[B115] Yadav N. Srivastava P. (2019). *In vitro* studies on gelatin/hydroxyapatite composite modified with osteoblast for bone bioengineering. Heliyon 5 (5), e01633. 10.1016/j.heliyon.2019.e01633 31193071 PMC6514539

[B116] Yarger J. L. Cherry B. R. van der Vaart A. (2018). Uncovering the structure–function relationship in spider silk. Nat. Rev. Mater. 3 (3), 18008. 10.1038/natrevmats.2018.8

[B117] Yu X. Huang J. Wu C. Zhang W. (2025). Biocompatible autonomous self-healing PVA-CS/TA hydrogels based on hydrogen bonding and electrostatic interaction. Sci. Rep. 15 (1), 1893. 10.1038/s41598-025-85298-3 39805869 PMC11730298

[B118] Zhang J. Shi X. Chen X. Huo X. Yu Z. (2021). Microbial‐induced carbonate precipitation: a review on influencing factors and applications. Adv. Civ. Eng. 2021 (1), 9974027. 10.1155/2021/9974027

[B119] Zhang J. Baeckens S. Damme R. V. Wanieck K. (2025). Overlooked sources of inspiration in biomimetic research. Sci. Rep. 15. 10.1038/s41598-025-11703-6 PMC12263887 40664831

[B120] Zhao B. Li N. K. Yingling Y. G. Hall C. K. (2016). LCST behavior is manifested in a single molecule: elastin-like polypeptide (VPGVG)n. Biomacromolecules 17 (1), 111–118. 10.1021/acs.biomac.5b01235 26595324

